# Identification of potent schistosomicidal compounds predicted as type II-kinase inhibitors against *Schistosoma mansoni* c-Jun N-terminal kinase SMJNK

**DOI:** 10.3389/fpara.2024.1394407

**Published:** 2024-04-26

**Authors:** Bernardo P. Moreira, Sandra G. Gava, Simone Haeberlein, Sophie Gueye, Ester S. S. Santos, Michael H. W. Weber, Tigran M. Abramyan, Christoph G. Grevelding, Marina M. Mourão, Franco H. Falcone

**Affiliations:** ^1^Institut für Parasitologie, Biomedizinisches Forschungszentrum Seltersberg (BFS), Justus Liebig Universitaet Giessen, Giessen, Germany; ^2^Grupo de Pesquisa em Helmintologia e Malacologia Médica, Instituto René Rachou, Fundação Oswaldo Cruz – Fiocruz, Belo Horizonte, Brazil; ^3^Polytech Angers, Université d’Angers, Angers, France; ^4^Rechenkraft.net e.V., Marburg, Germany; ^5^Atomwise, San Francisco, CA, United States

**Keywords:** JNK inhibitors, *Schistosoma mansoni*, kinase type-II inhibitors, c-Jun N-terminal kinase, kinase protein inhibitors

## Abstract

**Introduction:**

Schistosomiasis has for many years relied on a single drug, praziquantel (PZQ) for treatment of the disease. Immense efforts have been invested in the discovery of protein kinase (PK) inhibitors; however, given that the majority of PKs are still not targeted by an inhibitor with a useful level of selectivity, there is a compelling need to expand the chemical space available for synthesizing new, potent, and selective PK inhibitors. Small-molecule inhibitors targeting the ATP pocket of the catalytic domain of PKs have the potential to become drugs devoid of (major) side effects, particularly if they bind selectively. This is the case for type II PK inhibitors, which cause PKs to adopt the so-called DFG-out conformation, corresponding to the inactive state of the enzyme.

**Methods:**

The goal was to perform a virtual screen against the ATP pocket of the inactive JNK protein kinase. After virtually screening millions of compounds, Atomwise provided 85 compounds predicted to target c-Jun N-terminal kinase (JNK) as type II inhibitors. Selected compounds were screened *in vitro* against larval stage (schistosomula) of *S. mansoni* using the XTT assay. Adult worms were assessed for motility, attachment, and pairing stability. Active compounds were further analyzed by molecular docking against SmJNK.

**Results:**

In total, 33 compounds were considered active in at least one of the assays, and two compounds were active in every *in vitro* screening assay. The two most potent compounds presented strong effects against both life stages of the parasite, and microscopy analysis showed phenotypic alterations on the tegument, in the gonads, and impairment of cell proliferation.

**Conclusion:**

The approach to screen type II kinase inhibitors resulted in the identification of active compounds that will be further developed against schistosomiasis.

## Introduction

Schistosomiasis is a neglected tropical disease caused by trematodes of the genus *Schistosoma*. It is estimated that at least 251.4 million people needed preventive treatment in 2021, and transmission of the disease has been reported in 78 countries (WHO, 2023). While responsible for around 280,000 deaths each year, schistosomiasis is the second most important parasitic disease after malaria in terms of morbidity (LoVerde, 2019).

The disease is mainly caused by three different species, *Schistosoma mansoni, Schistosoma haematobium*, and *Schistosoma japonicum*, which share similarities within most of their complex life cycles. Miracidia, which hatch from eggs, infect an intermediate host (freshwater snail). Inside the snail, they develop via sporocysts stages to a free-living swimming larval stage – cercariae – that shed from snails to infect humans or other animals as definitive hosts. Schistosomula then develop to their adult form while migrating inside the host in order to reach the mesenteric veins of the gut (*S. mansoni* and *S. japonicum*) or venous plexus of the bladder (*S. haematobium*). Once there, they lay eggs that are released into the environment, allowing the continuation of the life cycle and transmission of the infection (Colley et al., 2014). Schistosomes are the only mammalian trematodes that have separate male and female sexes and exhibit sexual dimorphism in the adult stage. Besides, this parasite depends on pairing in order for the female to reach a mature reproductive stage (Popiel and Basch, 1984; Kunz, 2001; LoVerde et al., 2009). Once paired, the female can produce from a few hundred to thousands of eggs per day (Cheever et al., 1994; Burke et al., 2009).

Developed in the 1970s, praziquantel (PZQ) has become the main drug to treat and control schistosomiasis because of its high efficacy and low cost, besides the very few transient side effects (Gönnert and Andrews, 1977). However, due to its massive and repeated use on a large number of individuals over the last three decades, the emergence of parasite resistance to the drug is very much feared (Wang et al., 2012; Valentim et al., 2013). Besides, PZQ has shown limited effectiveness against juvenile stages of the parasite *in vivo* (Kabuyaya et al., 2018; Aboagye and Addison, 2023; Waechtler et al., 2023). It is therefore necessary to focus on finding new drug treatments, alternative or complementary to PZQ, against this disease (Mäder et al., 2018; Le Clec’h et al., 2021; Nogueira et al., 2022; Caldwell et al., 2023). As the search for alternative drug therapy against schistosomiasis intensifies, so does the search for new drug targets in this organism. Due to the pivotal role of protein kinases (PKs) in cancer, successful targeting of this family of proteins and drug development efforts have led to the FDA approval of 80 small-molecule kinase inhibitors as of today (Roskoski, 2024). Hence, a lot of attention has been placed on this protein family in order to find new therapeutic targets against schistosomes (Andrade et al., 2011; Beckmann, 2012; Gelmedin et al., 2015; Stroehlein et al., 2015; Grevelding et al., 2018; Li et al., 2019; Wu et al., 2021; Moreira et al., 2022).

The research on schistosomes shows that Mitogen-Activated Protein Kinases (MAPKs) play important roles in the motility, development, survival, and reproduction of these parasites (Wang et al., 2006; Ressurreição et al., 2011; Andrade et al., 2014; Avelar et al., 2019). Considering the importance of MAPKs in the context of this parasite, exploring the possibilities of targeting c-Jun N-terminal protein kinase (JNK) is paramount, especially when the structures of PKs are well characterized. Here, we targeted the human JNK2 by *in silico* and *in vitro* screening. JNK is a serine/threonine kinase belonging to the MAPK subfamily, a group of evolutionarily conserved cellular regulatory molecules that convert extracellular stimuli in the form of phosphorylation cascades to intracellular responses. SmJNK knock-down by RNA interference (RNAi) revealed its involvement in parasite maturation and survival within the host, impacting oviposition and altering the expression of genes related to important cellular processes (Andrade et al., 2011; Gava et al., 2019). Our last study has identified inhibitors predicted to target SmJNK – in addition to other *S. mansoni* MAPK family members (Moreira et al., 2022), with no other drug screening against this specific target being reported. Whereas in our previous study, we explored the ATP-binding site of SmJNK and looked specifically at type I-inhibitors of SmJNK, in the present study we have focused on type II-inhibitors.

Our current analysis focused on the catalytic cleft located between both lobes of the kinase domain (KD). This main cleft is where the ATP binds, which is the major focus of kinase inhibitor development. Although the majority of PK inhibitors (PKI) compete with ATP for the same pocket, several other important regions can participate in the formation of this catalytic cleft. These include, for instance, the glycine-rich loop (G-rich loop), the αC-helix in the N-terminal domain, the gatekeeper residue (preceding the hinge region), and the conserved lysine residue (β-sheet III in N-terminal lobe), which forms a salt bridge with the conserved glutamate residue from the αC-helix and Asp-Phe-Gly (DFG) motif. The flexibility of these regions allows for different conformations, thereby providing a large variety of options for inhibitor binding. Furthermore, active and inactive kinase conformations can be defined relative to the positioning of the DFG motif. In the active state or DFG-in conformation, the phenylalanine (F residue) is buried inwards towards the hydrophobic pocket in the groove between the two kinase lobes and the αC-helix is positioned closer to the catalytic cleft. Conversely, in the inactive state or DFG-out conformation, a 180°-flip reorients the F residue outwards, with a looser attachment or displacement of more than 10 Å of the αC-helix.

Different classes of PKIs bind to this cleft in a different manner and interact with different residues within the pocket. Kinase inhibitors (KI) were first classified by Dar and Shokat (2011) into types I, II, and III. Whereas type I molecules bind within the ATP pocket of active kinases (DFG-in conformation), type II inhibitors bind to the inactive state of a kinase ATP pocket under a so-called DFG-out conformation. This class is considered ATP competitive with a reversible binding mode. Small-molecule inhibitors targeting the ATP pocket of the catalytic domain of PKs have potential to become drugs devoid of (major) side effects, particularly if they bind selectively. This is the case for type II PK inhibitors. With this in consideration, the goal of this study was to predict type II inhibitors of SmJNK by applying a virtual screening against the ATP pocket of the human JNK2 in a state (DFG-out conformation) where selectivity has more often been observed, addressing potential kinase selectivity issues. Subsequently, predicted type II kinase inhibitors were selected and compounds were then screened *in vitro* against schistosomula and adult worms of *S. mansoni*.

## Methods

### Parasites

Freshwater snails (*Biomphalaria glabrata*) served as the intermediate hosts for the ST (Liberia) strain of *S. mansoni* (Grevelding et al., 1997). As final hosts, golden hamsters (*Mesocricetus auratus*, Janvier, France) at the age of 8 weeks were infected by the paddling method using up to 1,750 cercariae per hamster (Dettman et al., 1989). Worms were collected by hepatoportal perfusion 46 days (d) post-infection (p.i.). Additionally, intermediate host snails (*Bulinus truncatus subsp. truncatus*, NR-21965) shedding an Egyptian strain of *S. haematobium* were provided by the NIAID Schistosomiasis Resource Center for distribution through BEI Resources, NIAID, and NIH. In this case, hamsters were infected with 1,500 cercariae and adult worms were collected at 20 weeks p.i., as described above. For regular maintenance *in vitro*, worms were cultured in M199 medium (Merck, Germany) supplemented with 10% Newborn Calf Serum (NCS, Merck), 25-mM HEPES (ThermoFisher Scientific, USA), and 1% ABAM solution (100-units/mL penicillin, 100-µg/mL streptomycin, and 250-µg/mL amphotericin B; Merck) at 37°C in a 5% CO_2_ atmosphere (Grevelding et al., 1997).

*S. mansoni* cercariae were shed from snails and counted before being subjected to mechanical transformation to schistosomula. Cercariae were mechanically transformed into schistosomula according to Milligan and Jolly (2011) with modifications. After passing samples through the syringes, all subsequent washes were carried out with M199 medium without phenol red (ThermoFisher Scientific), supplemented with 2% Penicillin/Streptomycin (PenStrep, 100 U/mL, ThermoFisher Scientific) and 2% heat-inactivated Fetal Bovine Serum (FBS, Gibco, USA). Lastly, newly transformed schistosomula were incubated overnight in supplemented M199 medium at 37°C and 5% CO_2_ for recovery before *in vitro* screening.

For WormAssay analysis, adult worms from *S. mansoni* LE strain (Belo Horizonte) were obtained by perfusion from six-week-old female Golden hamsters (*M. auratus*) 45 days after subcutaneous infection with 400 cercariae each, as previously described (Pellegrino and Siqueira, 1956; Tavares and Mourão, 2021). Cercariae were acquired from the Mollusk rearing facility “Lobato Paraense” of René Rachou Institute – Fiocruz Minas, where the parasite life cycle is maintained using *B. glabrata* as the intermediate snail host. Eight male and female worms, separately, were cultured in 24-well polystyrene plates in RPMI 1640 medium (Merck) supplemented with 2% PenStrep (100 U/mL, Gibco) and 10% heat-inactivated FBS (Gibco) and incubated at 37°C, 5% CO_2_, and 95% humidity in a CO_2_ incubator.

### Kinase structures and *in silico* construction of a *S. mansoni* JNK (Smp_172240) model in the inactive (DFG-out) kinase conformation

The crystal structure of human JNK2 was retrieved from PDB database (PDBID: 3PNC) (Kuglstatter et al., 2010), and a machine learning-based model of *S. mansoni* JNK (Smp_172240) was obtained through AlphaFold2 prediction (Jumper et al., 2021; Varadi et al., 2022), as stored in Uniprot dated on 2022/06/01 (https://alphafold.ebi.ac.uk/entry/A0A3Q0KT26). Six complementing single-template (PDB ID: 3NPC) approaches were pursued to construct an *in silico* homology model for SmJNK in its inactive (DFG-out) kinase conformation using the following advanced modeling environments: SwissModel (Waterhouse et al., 2018), I-Tasser (Roy et al., 2010), Phyre2 (Kelley et al., 2015), RobettaCM (Song et al., 2013), TopModel (Mulnaes et al., 2020, 2021), and EasyModel (Arab and Dantism, 2023; based on MODELLER, Webb and Sali, 2016). In a second manual approach, dual-template homology modeling was performed in the MODELLER-based EasyModel software environment using full-length JNK2 (DFG-out) and the AlphaFold2 *S. mansoni* JNK model (DFG-in but deprived of the following DFG loop-comprising peptide: DFGLARTAGDSFLMTPYVVT) as templates. All resulting *S. mansoni* JNK models (DFG-out conformation) were AMBER-relaxed employing standard AlphaFold2 minimization settings on a T4 GPU as part of the Colabfold Jupyter notebook suite (Mirdita et al., 2022) and non-relaxed and AMBER-relaxed models were subjected to SwissModel’s detailed comparative structure assessment tool (Waterhouse et al., 2018). As a result, AMBER-relaxation consistently improved the model quality and the AMBER-relaxed SwissModel-generated SmJNK turned out to be the highest quality model, which was used for molecular docking. It should be noted that, likely due to the intrinsic properties of the machine learning model, manual AlphaFold2-based generation of an inactive state (DFG-out) SmJNK kinase model was impossible even when using exclusively JNK2 as the sole modeling template in Colabfold.

### *In silico* screening and compounds

The virtual screen of millions of commercially available chemical compounds was performed by Atomwise (California, USA) using AtomNet^®^ (Wallach et al., 2015; Stafford et al., 2022), a deep learning neural network for structure-based drug design and discovery platform. The computational screening protocol is described in detail elsewhere (The Atomwise AIMS Program, 2024). Briefly, we started with large commercially available compound libraries from MCule (mcule.com) and Enamine (enamine.net). The compounds were filtered using the Eli Lilly medicinal chemistry filters (Bruns and Watson, 2012) and removed potential false positives, such as aggregators, autofluorescers, and PAINS (Baell and Holloway, 2010). The filtered library was then virtually screened against the human JNK2 crystal structure (PDB ID 3NPC) in the DFG-out conformation, excluding molecules with greater than 0.5 Tanimoto similarity in ECFP4 space (Rodgers and Hahn, 2010) to any known binders of the target and its homologs within 70% sequence identity. To ensure diversity of compounds for experimental validation the top 30,000 virtual hits were clustered using the Butina algorithm (Butina, 1999), with ECFP4 fingerprints allowing a maximum 0.35 Tanimoto similarity between clusters. The top 85 diverse compounds were quality controlled by LC-MS to ensure >90% purity and purchased. All compounds were reconstituted to 20 mM in 100% DMSO (Merck). A datasheet containing all the information regarding the 85 selected compounds is provided in **Supplementary Table S1**. The pharmacokinetic parameters encompassing absorption, distribution, metabolism, excretion, and toxicity (ADMET) of the compounds were predicted using the pkCSM web server (http://biosig.unimelb.edu.au/pkcsm/) (Pires et al., 2015). Subsequently, the quantitative data obtained were converted into binary data based on reference values set by the tool. Then, hierarchical clustering of the binary data was carried out employing Euclidean distances, using the ComplexHeatmap package (v.2.16.0) (Gu et al., 2016) in R (v.4.3.2) (R Core Team, 2023). Additionally, a pkCSM score was calculated based on 29 predicted ADMET features, following the criteria outlined by Souza Silva et al. (2021). Furthermore, we used SwissADME (http://www.swissadme.ch) to check drug-likeness and medicinal chemistry friendliness features of compounds (Daina et al., 2017).

### Targeted molecular docking, docking data analysis and molecular visualizations

Molecular docking was performed utilizing BINDSURF (an Autodock Vina derivative) as implemented in Achilles (Sánchez-Linares et al., 2012) according to the instructions provided by the developers. Briefly, the ligand molecules were extracted as 3D structure files (.mol2 format) from the ZINC database (https://zinc.docking.org/) or PubChem (https://pubchem.ncbi.nlm.nih.gov/), checked for correct conformation, stereochemistry and pH-dependent protonation states using Chimera 1.1.6 (Pettersen et al., 2004) or ChimeraX 1.6 (Pettersen et al., 2021) and subjected to the docking pipeline after conversion to.mol2 file format (where required). The generated SmJNK-ligand complex structure files were initially inspected using the Achilles result web interface and then subjected to in-depth compound-binding analyses employing PLIP (Adasme et al., 2021). Molecular visualizations were prepared in Chimera 1.16 (Pettersen et al., 2004), ChimeraX 1.6 (Pettersen et al., 2021) or PyMOL 2.5 (Delano, 2002).

### XTT schistosomula viability assay

Schistosomula were screened against the selected compounds using the XTT viability assay according to Aguiar et al. (2017). In summary, 400 newly transformed schistosomula per well were plated in 96-well plates in 200 µL of M199 without phenol red (Merck) supplemented with 100 U/mL penicillin and 100 μg/mL streptomycin. Compounds were added at a final concentration of 20 µM, and plates were incubated at 37°C and 5% CO_2_. Negative controls were incubated with 2% DMSO, and heat-killed parasites (previously incubated at 65°C for 10 min) were used as positive controls. The tetrazolium salt XTT (2,3-Bis(2-methoxy-4-nitro-5-sulfophenyl)-2H-tetrazolium-5-carboxanilide inner salt, Merck) and enhancer phenazine methosulfate PMS (Merck) were prepared as described in Aguiar et al. (2017) and filtered solutions were stored at -20°C until use. After 48 h of incubation with the compounds, 40 µL of XTT : PMS mix (50:1 ratio) were added to each well. 24 h after the addition of XTT : PMS mixture to each well, absorbance readouts at 450 nm and 690 nm (reference wavelength) were performed using a CLARIOstar Plus multi-mode microplate reader (BMG Labtech, Offenburg, Germany). The percentage of schistosomula viability was calculated as described by Aguiar et al. (2017), taking into account the absorbance values from positive and negative controls. Experiments were repeated at least three times for each compound.

### *In vitro* screening of adult worms

One day after perfusion, the adult worm stage of *S. mansoni* was screened against the selected compounds and the following parameters were analyzed at 24, 48, and 72 h of incubation: worm pairing status, motility, attachment, alternative phenotypes, and egg count. In total, five worm pairs were added in each well and two independent experiments were performed for each compound. Compounds were added to a final concentration of 20 µM, and 0.4% DMSO was used as control. The supplemented medium was changed every day followed by the addition of compounds. Worms were analyzed under an inverted microscope attached to a camera (Zeiss Primovert - Axiocam 208 color, Oberkochen, Germany). The pairing status was expressed as the percentage of paired and unpaired couples at the specified time points. A motility score was assigned to each well by comparing motility of worms in wells with test compounds to the solvent control: a score of '0' was given to each worm/couple when no activity was observed after 20 seconds of observation; '1' for minimal activity with only residual movements of head and tail regions; '2' when reduced activity with weak and slow movements were observed; '3' when normal activity was observed; and '4' when hyperactivity was observed. An average score of all the worms per well was calculated to obtain a motility score. For this average, if worms were still in couples, the motility score counted double (1 couple = 2 worms). In parallel, worms were assessed for attachment to the well plate and alternative phenotypic effects were observed. For adult worm stages of *S. haematobium*, only worm motility was evaluated, as described above. Since mostly male *S. haematobium* can be harvested by hepatoportal perfusion, only males were used for *in vitro* experiments.

For a more extended analysis of motility and to assess the activity of compounds depending on parasite sex, the movement of male and female adult worms of *S. mansoni* was recorded daily for 1 min and 30 s over a period of 10 days using a Canon EOS REBEL T5 camera and the WormAssay software (Marcellino et al., 2012). For this, parasites, male and female separately, were exposed to 20 µM of compounds for 24 h, and subsequently the supplemented medium was changed every other day, without the addition of any compound. Compounds were considered active when causing at least 50% of motility alteration relative to DMSO.

### Confocal laser scanning microscopy of adult worms

Effects of *in vitro* culture with selected compounds on cell proliferation were assessed by EdU proliferation assay. To this end, EdU was added to the worms in a final concentration of 10 µM after 24 h of compound exposure (for compound B08: after 4 h). The worms were fixed with 4% paraformaldehyde after another 24 h of culture, stained with the Click-iT Plus EdU Alexa Fluor 488 imaging kit (Thermo-Fisher Scientific) and counterstained with Hoechst 33342 as previously described (Kellershohn et al., 2018). Morphological effects on organ structures were assessed by fixing worms in AFA (66.5% ethanol, 1.1% paraformaldehyde, 2% glacial acetic acid) and staining with CertistainH carmine red (Merck, Germany) as previously described (Beckmann and Grevelding, 2010). Stained worms were imaged using a TSC SP5 inverse confocal laser-scanning microscope (CLSM) (Leica Microsystems, Wetzlar, Germany). AlexaFluor488 and carmine red were excited using an argon-ion laser at 488 nm, and Hoechst at 405 nm. Optical section thickness and background signals were defined by setting the pinhole size to 1 Airy unit in the Leica LAS AF software. A line average of four was applied for all recordings.

### Statistical analysis

Statistical analysis was performed in GraphPad Prism version 8.0.0 for Windows (GraphPad Software, La Jolla California USA). Kruskal-Wallis test with uncorrected Dunn’s test was used to analyze schistosomula XTT assays. Two-way ANOVA with uncorrected Fisher’s LSD test was used to analyze data regarding adult worm motility and pairing. A mixed-effects model, using the restricted maximum likelihood method was employed to analyze WormAssay data. *Post hoc* analysis was conducted using the uncorrected Fisher’s LSD test. Differences were considered significant when p-value < 0.05.

## Results

### Virtual screening predicts type II inhibitors against SmJNK

**Figure 1:**
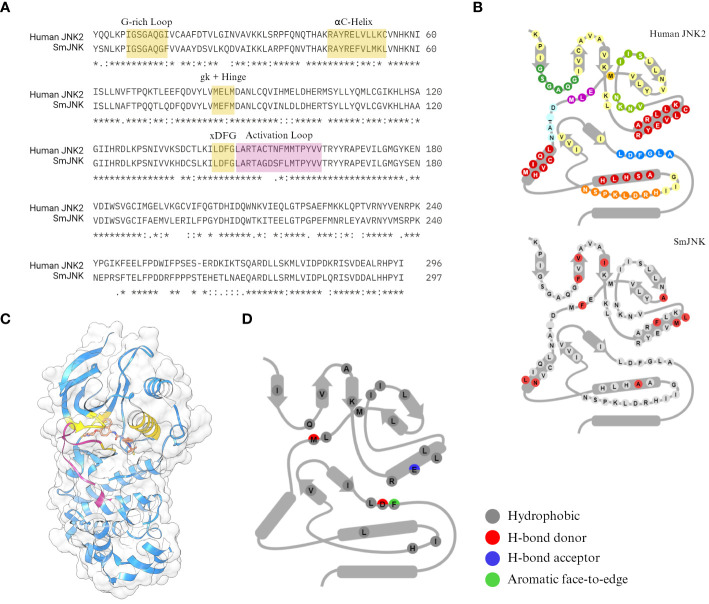
Sequence conservation between the SmJNK and JNK2. **(A)** Sequence identity between the human JNK (JNK2) and *S. mansoni* JNK (SmJNK) PKD was analysed by sequence alignment in Clustal Omega (Sievers et al., 2011). Conserved domains for kinase-ligand interaction were highlighted in yellow (G-rich loop, αC-helix, gatekeeper (gk) residue, hinge, and DFG motif. Activation loop is highlighted in magenta. **(B)** Schematic representation of the 85 amino acid residues from KLIFS database (Van Linden et al., 2014) of both kinases. The differences in SmJNK compared to the human JNK are highlighted in red. **(C)** Ribbon and surface representation of the crystal structure of JNK2 (blue) bound to BIRB796 (orange) (Kuglstatter et al., 2010). Domains highlighted in **(A)** are shown here in the same colors. **(D)** Schematic representation of kinase-ligand interactions of JNK2 and BIRB796 among the KLIFS residues.

The human c-Jun N-terminal kinase JNK2 (PDB ID: 3NPC) was selected by Atomwise as a template for the virtual screening. JNK2 has great coverage of the protein sequence and high sequence identity to SmJNK (73.2% of the PKD), with highly conserved motifs for ligand interaction (**Figure 1A**). Among the many differences in amino acid conservation throughout the PKD of JNK2 and SmJNK, 11 residues differ within the so-called 85 KLIFS residues (Van Linden et al., 2014) (**Figure 1B**), which are directly responsible for the binding pose of kinase inhibitors. More importantly, the crystal structure of JNK2 was solved with a type-II inhibitor, BIRB796 (Kuglstatter et al., 2010) (**Figure 1C**). The docking of BIRB796 within the pockets of the JNK2 kinase is characterized by multiple hydrophobic and polar interactions with key residues that often participate in the binding patterns of type II inhibitors (**Figure 1D**). Hence, the goal was to perform the virtual screen of JNK in its inactive state (DFG-out conformation), where selectivity has more often been observed, in order to aid with potential kinase selectivity issues.

After a virtual screening of several millions compounds against JNK2, Atomwise provided 85 compounds that were predicted to bind as type II inhibitors to JNK. Among those, 82 complied with Lipinski’s Rule of Five, with the exception of A02, F01, and G02. One compound (D09) presented pan-assay interference (PAINS) and 39 compounds satisfied leadlikeness criteria according to SwissADME analysis (**Supplementary Table S1**). After reconstitution in 100% DMSO, which worked as a solvent for all compounds, only compound A11 presented low solubility, but it was kept in the analysis.

### Two compounds have strong antischistosomal activity against adult and larval stages

**Figure 2:**
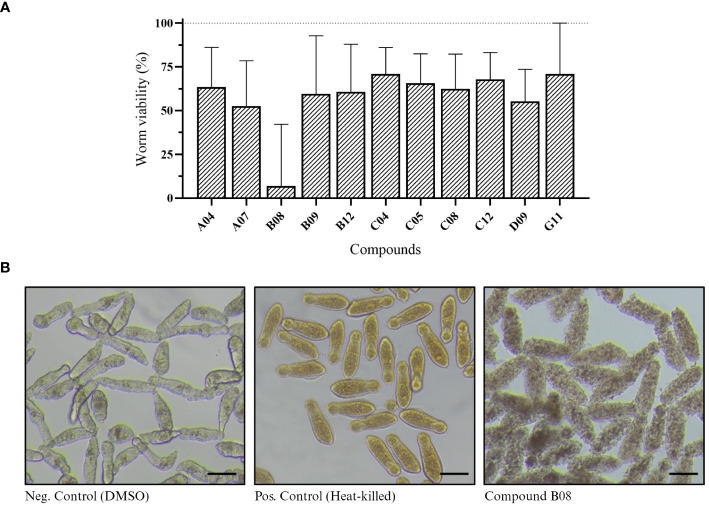
*In vitro* screening against schistosomula. **(A)** Worm viability measured by XTT assay and calculated as described in Methods. Bars represent viability related to the negative (DMSO, 100%) and positive controls (heat-killed, 0%). Experiments were performed with 400 worms per well (n ≥ 3). **(B)** Light microscopy (bright-field) of schistosomula in 96-well plates after 72 hours of incubation with 20 µM of compound B08. DMSO and heat-killed controls are shown. Scale bar: 100 µm.

Next, we performed XTT assay to assess the viability of *in vitro* schistosomula when exposed to the compounds (Aguiar et al., 2017). Of 85 compounds, 11 presented a significant effect on schistosomula (**Supplementary Figure S1**). The absorbance values of active compounds were transformed to show worm viability in a bar chart graph (**Figure 2A**). After 72 h of incubation with 20 µM, compound B08 presented the strongest effect against schistosomula, causing a decrease in viability of approximately 94.1%. Compared to the negative control and heat-killed parasites, the effect of compound B08 was characterized by a complete degeneration of the worm body (**Figure 2B**).

**Figure 3:**
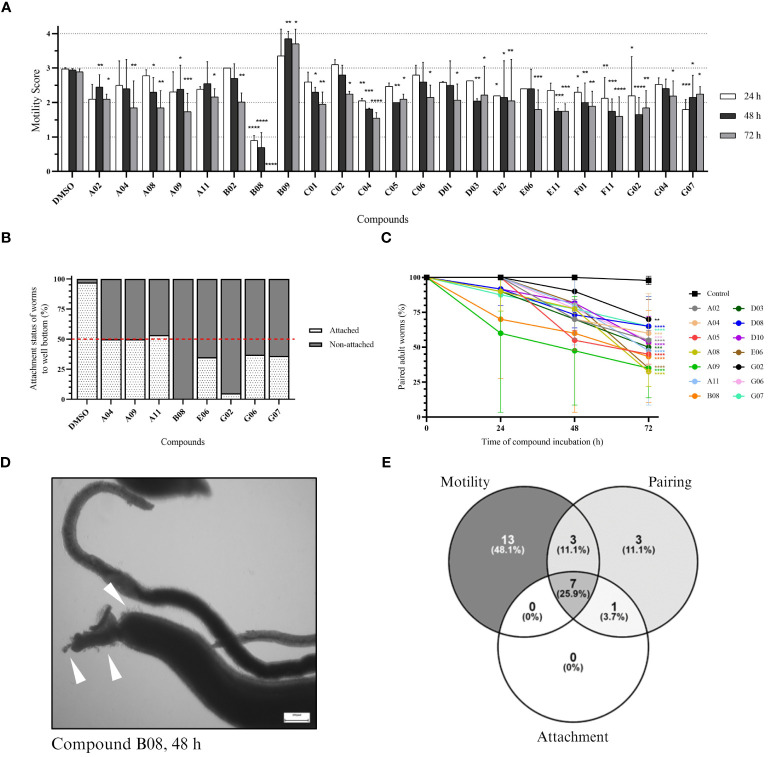
*In vitro* screening against adult worms. **(A)** Bar representation of the motility score of active compounds at 24-, 48-, and 72-hour timepoints after compound incubation. Worms were observed daily, and a score was given to each worm (or pair of worms) as described in the Methods section. Plotted scores (mean + SD) represent two independent experiments and two-way ANOVA with uncorrected Fisher’s LSD was performed [p-value < 0.0332 (*); < 0.0021 (**); < 0.0002 (***); < 0.0001(****)]. **(B)** Bar representation in the percentage of the attachment status of worms after 72 hours of incubation. Plotted scores (mean) represent two independent experiments. **(C)** Points-and-line graph representation of the percentage of paired worms at each observed timepoint. Plotted scores (mean + SD) represent two independent experiments and two-way ANOVA with uncorrected Fisher’s LSD was performed [p-value < 0.0332 (*); < 0.0021 (**); < 0.0002 (***); < 0.0001(****)]. **(D)** Light microscopy (bright field) of adult worms (male and female) in 12-well plates after 48 hours of incubation with 20 µM of compound B08. White arrowheads indicate tegumental damage. Scale bar: 200 µm. **(E)** Venn’s diagram of the number of active compounds within the three parameters analysed for adult worms.

The same set of compounds was screened against adult worms of *S. mansoni*. Here, three parameters were monitored: worm motility, attachment, and pairing status. Regarding worm motility, 26 compounds caused a significant change in parasite motility at 48 and/or 72 h after incubation (**Supplementary Figure S2**). The 23 compounds that show a significant effect after 72 h of incubation are depicted in **Figure 3A**. While 22 compounds caused a decrease in parasite movement, one compound (B09) caused an inverse effect, with parasites displaying hyper-activity when compared to the control. Compound B08 caused the most severe effect in adult worms. The high potency of the compound could be immediately detected by the lack of parasite movement since the first time point analyzed, leading to the death of all parasites after 72 h of treatment. We also observed the effect of drugs on the attachment of worms to the bottom of the well. Healthy moving worms are mostly attached to the bottom of the well by their ventral sucker. In total, seven compounds were considered active (**Supplementary Figure S3**; **Figure 3B**). Whereas six compounds caused at least 50% of worm detachment, compound A11 did not reach that threshold, however it was included as active due to the observed strong effect (46.7%). Compared to the control, compounds B08 and G02 presented the most severe effects, with 100% and 95% detachment, respectively. Lastly, we observed the pairing status of adult worm couples. In total, 14 compounds were considered active in causing the separation of worm couples (**Supplementary Figure S4**; **Figure 3C**). The most active compounds were A08, A09, and E06, separating 67.5%, 65%, and 65% of couples, respectively. In contrast to the strong effects observed for compound B08 in motility and attachment parameters, at first view B08 caused only a moderate effect on pairing stability. However, we think that the observed separation level of 50% is a direct consequence of a quick killing of the worms, of which many died before they could split. Morphological inspection exhibited severe tegumental damage as early as 48 h of incubation with B08 (**Figure 3D**). A summary of the number of active compounds is presented by Venn’s diagram, showing that seven compounds (A04, A09, A11, B08, E06, G02, and G07) can be considered active in all three observed parameters (**Figure 3E**). Additionally, two of these seven compounds (A04 and B08) were also active against schistosomula.

### Compound activity differs depending on parasite species, strain, and sex

**Figure 4:**
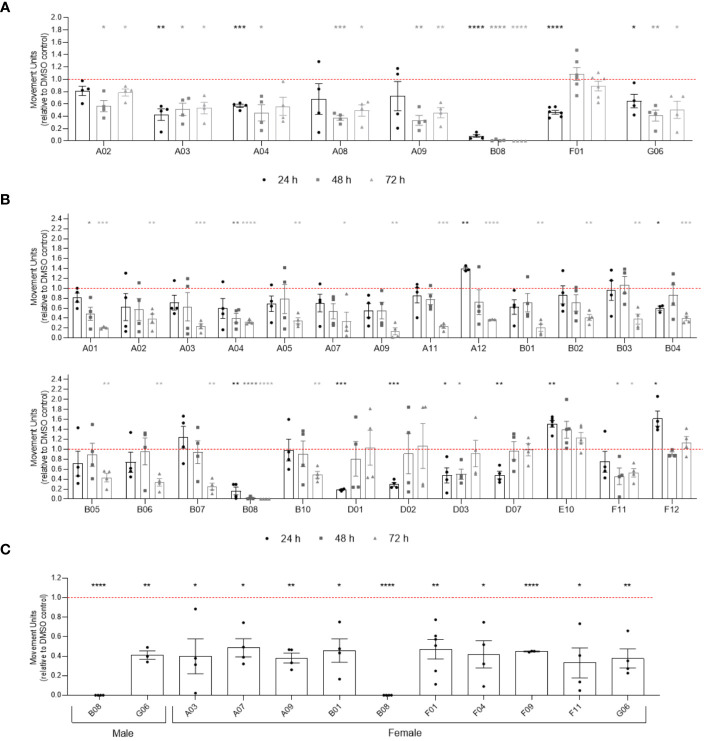
Activity of compounds related to sex difference in *in vitro* screening of adult worms. Bar representation of the mean of movement units of male **(A)** and female **(B)** adult worms after 24 h, 48 h and 72 h of exposure to compounds at 20 µM. **(C)** Compounds with prolonged activity after 10 days of exposure to compounds at 20 µM. The movement units of worms exposed to compounds were normalised relative to the movement units of worms exposed to the vehicle control DMSO 0.4% (red dotted line). Error bars are represented above the points. Statistical analyses using mixed-effects model are represented with asterisks above the points (*p < 0.05, **p < 0.01, ***p < 0.001 and ****p < 0.0001).

To verify if compounds exhibit differential activity depending on the sex of parasites, we performed WormAssay analyses, monitoring the motility of male and female parasites separately over 10 days after compound exposure (**Figure 4**; **Supplementary Figure S5**). Following 24, 48 or 72 h of compounds exposure, eight compounds were active in males (**Figure 4A**), while 25 were active against female adult worms (**Figure 4B**). After 10 days of incubation with compounds, only two compounds (B08 and G06) remained active in males, contrasting with ten (A03, A07, A09, B01, B08, F01, F11, F04, F09, and G06) in females that promoted motility reduction in the long term (**Figure 4C**). Notably, 19 compounds (A01, A12, B01-B04, B06, B07, B10, D01-D03, D07, E02, E10, F04, F09, F11, F12) were active in females only, while compound A08 was exclusively active in males. Additionally, while the WormAssay results mentioned above were performed using the LE strain of *S. mansoni*, worms from the ST (Liberia) strain were used in previous motility, attachment, and worm pairing assessment. We observed some discrepancy when comparing active compounds detected in motility analysis for both strains. For instance, some compounds (A01, A03, A05, A07, A12, B01, B03, B04, B05, B06, B07, B10, D02, D07, E10, and F12) that were active in the female LE strain did not present activity in couples of the ST (Liberia) strain during the 3 days of motility analysis, which may be attributed to the masking of the female motility phenotype by pairing with the male worm.

In order to assess for cross-species effects of the active compounds in adult *S. mansoni*, we also screened male *S. haematobium* adult worms against seven selected active compounds (A04, A09, A11, B08, E06, G02, G07) that showed strong activity in most assays. Motility assessment of adult *S. haematobium* showed that compounds A04, A09, and B08 were able to cause a significant reduction in the motility of the parasite after 48 and 72 h of incubation (**Supplementary Figure S6A**). Furthermore, compound B08 killed male *S. haematobium* worms, as observed in *S. mansoni* (**Supplementary Figure S6B**), indicating that compound B08 targets different pathogenic *Schistosoma* species.

Regarding the biochemical properties of active compounds, the pkCSM tool was employed to calculate a score based on the cumulative count of positive and negative ADMET features, with scores ranging from -4 to 15. Two of the active compounds presented scores equal or higher than PZQ (score 11). Compound D10 (score 13) was the highest pkCSM score, and it was active against adult worms (**Supplementary Figure S7**). However, it was only able to affect worm pairing. Compound D07, which scored equal to PZQ, was active against adult female worms, but only affecting motility after 10 days of drug exposure (**Supplementary Figure S7**). Compound D09, which was active against schistosomula, was the only compound that presented pan-assay interference (PAINS) (**Supplementary Table S1**). This indicates that all compounds, except for D09, are not considered promiscuous and should not affect assays read-out. Moreover, among the active compounds, only A02, F01, and G02 do not comply with the Lipinski’s Rule of Five.

### Compound B08 affects various tissues and impairs cell proliferation in treated worms

**Figure 5:**
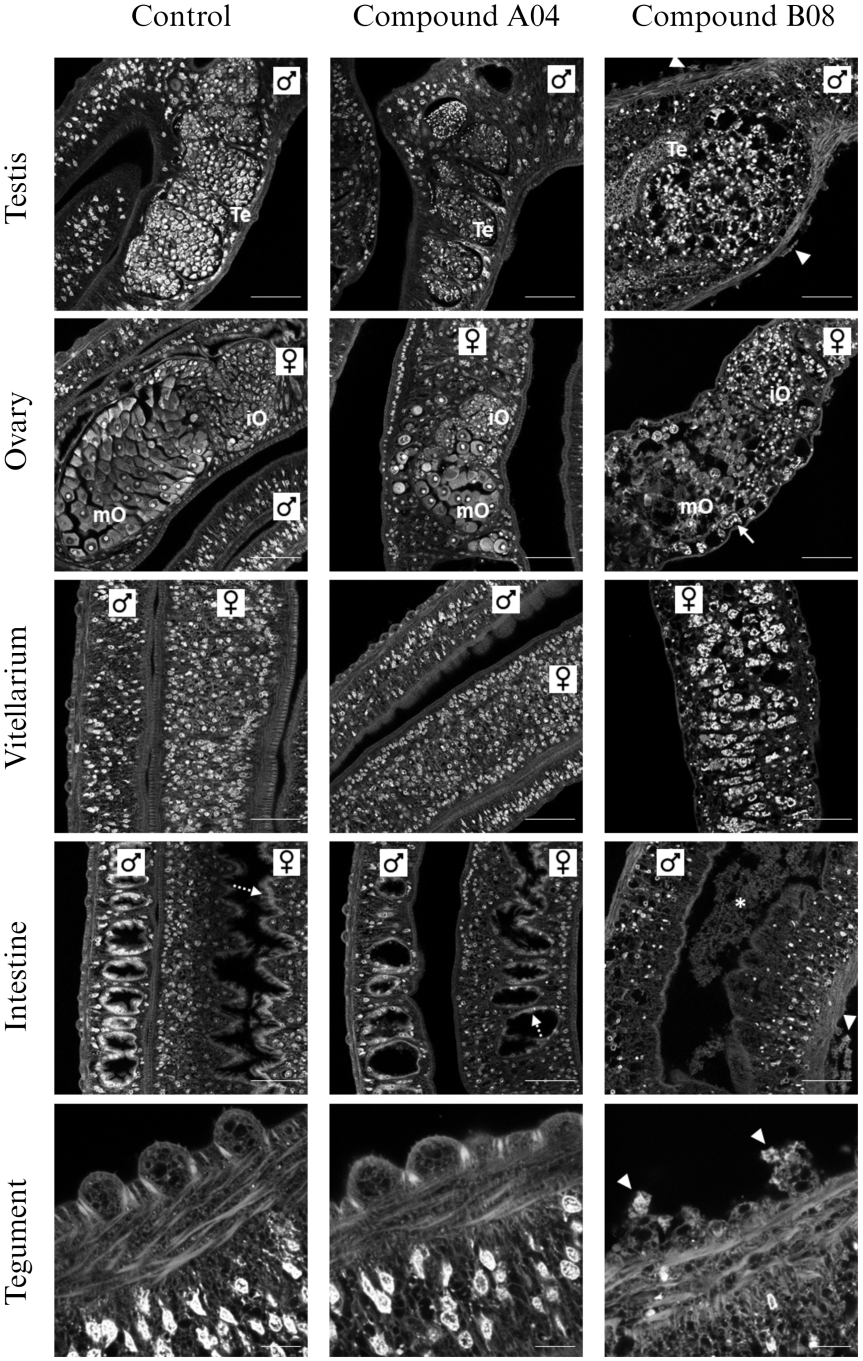
Effects of compounds A04 and B08 on the tissue structure of male and female adult worms. *S. mansoni* couples were exposed to A04 or B08 (both 20 µM) or DMSO as negative control and stained with carmine red. Both compounds affected the ovary regarding size or internal structure (iO, immature oocytes; mO, mature oocytes). B08 also disrupted the testicular lobes (Te, testes) in male worms and caused a loss of immature vitellocytes in the female vitellarium. Here, mostly mature S4 cells with prominent lipid droplets were left (also note the accumulation of S4 cells in the vitelloduct (ovary), marked by an arrow). Furthermore, B08 damaged the tegument with disruption of the tubercles of male worms (marked by triangles) and the gut (intact gastrodermis marked by dashed arrows; degraded tissue in gut lumen marked by *). Representative CLSM images of at least four worms per sex are shown. Scale bars: 50 µm (tegument) or 100 µm (others).

Since A04 and B08 caused strong impairment of adult worms, we performed CLSM to reveal possible reasons for the reduction of vitality (**Figure 5**). On the cellular and tissue level, worms treated with A04 for 72 h appeared comparable to control worms. Only the A04-treated females displayed ovaries of only about half of the size as in controls. Both areas of the ovary, containing stem cell-like oogonia and mature oocytes, were smaller. On the contrary, treatment with B08 for only 48 h severely disrupted the structure of almost every tissue (**Figure 5**). The gonadal tissues (testes, ovary, and vitellarium) displayed a patchy appearance with obvious cell loss. No intact mature oocytes were found within the ovaries. The vitellarium largely lacked immature vitelline cells, with only large S4 stage vitellocytes left, which were sometimes found accumulating in the vitelloduct. Epithelial tissues (gastrodermis and tegument) were in parts degraded.

**Figure 6:**
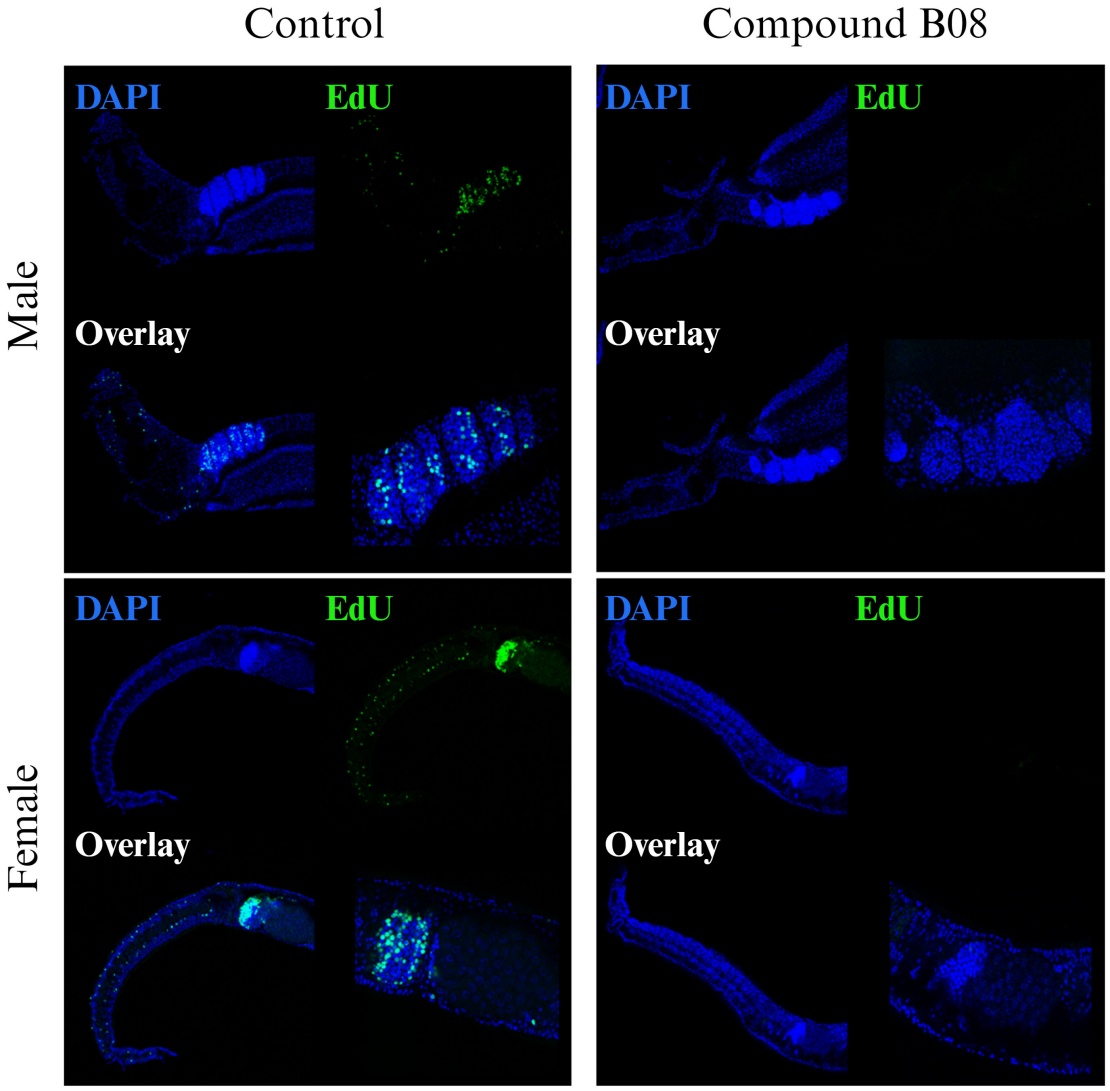
Compound B08 abolishes cell proliferation in adult worms. *S. mansoni* couples were exposed to B08 (20 µM) or DMSO as negative control and proliferating cells were visualized by EdU incorporation assay. Representative CLSM images from four worms per sex are shown (green: proliferating cells; blue: cell nuclei). Scale bar represents 100 µm (overview) or 50 µm (magnification of gonads).

The tissue degeneration and especially the reduction in numbers of immature and maturing germline cells after B08 treatment led us to speculate on a defect in cell proliferation and/or differentiation. While the other six prioritized compounds did not affect EdU-positive staining in treated worms compared to control worms (**Supplementary Figure S8**), compound B08 abolished cell proliferation, as shown by the lack of EdU incorporation. Because of the fast action of compound B08 (**Figures 3A**, **4A**), we started the EdU culture after 4 hours of treatment. Compound B08 almost completely ablated EdU staining in both neoblasts (somatic stem cells) and germline stem cells (**Figure 6**) with just some neoblasts stained in one of several females (not shown). This further supports the strong and quick effect caused by this compound.

### Compounds A04 and B08 are predicted to bind preferentially inactive SmJNK (DFG-out)

**Figure 7:**
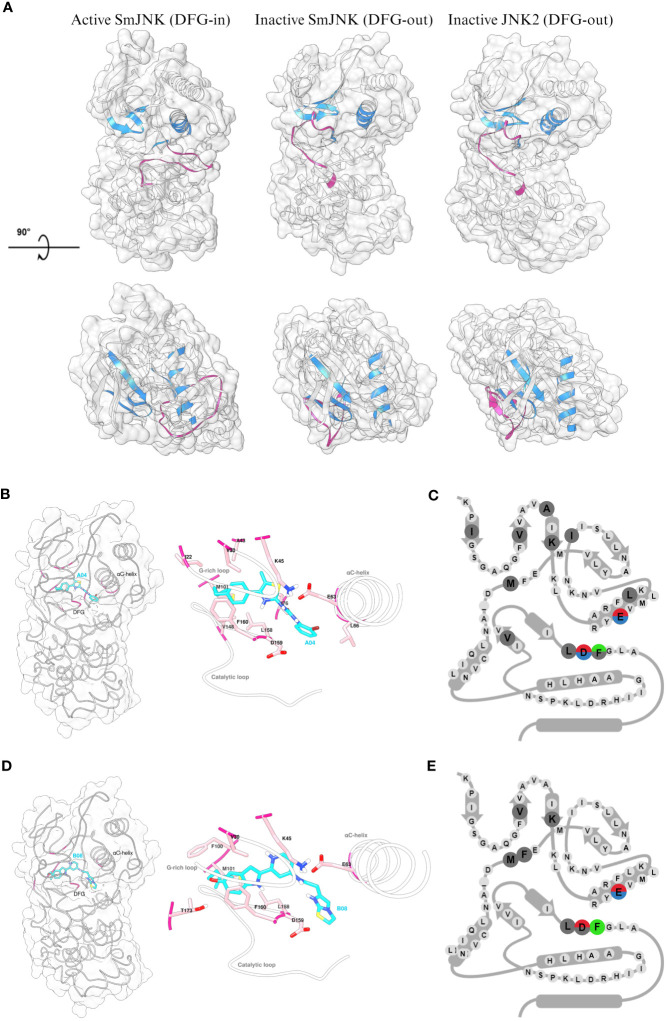
Detailed analysis of the interaction of inactive SmJNK with compounds A04 and B08. **(A)** Ribbon and surface representation (white) of the homology models of SmJNK in active (DFG-in) and inactive (DFG-out) conformation, and crystal structure of human JNK2 (DFG-out). G-rich loop, αC-helix, and DFG motifs are colored in blue. Activation loop is colored in magenta. **(B)** Left: ribbon and surface representation of inactive SmJNK (grey) and docked A04 (cyan) with interacting residues highlighted in magenta. Right: Detailed representation of the docking with labeled residues and their side chains in stick representation. G-rich loop, αC-helix, and the catalytic loop are shown in white. **(C)** Schematic representation of KLIFS residues showing the kinase-ligand interactions highlighted in **(B)**. **(D)** Left: ribbon and surface representation of inactive SmJNK (grey) and docked B08 (cyan) with interacting residues highlighted in magenta. Right: Detailed representation of the docking with labeled residues and their side chains in stick representation. G-rich loop, αC-helix, and the catalytic loop are shown in white. **(E)** Schematic representation of KLIFS residues showing the kinase-ligand interactions highlighted in **(D)**.

Since the Uniprot AlphaFold2 model of *S. mansoni* JNK represents the canonical active state (DFG-in) kinase conformation, two complementary approaches, automated and manual homology modeling, were employed to generate *S. mansoni* JNK models in the desired inactive (DFG-out) kinase conformation. Here, the goal was to generate *a S. mansoni* JNK model as close as possible to the original AlphaFold2 model currently stored in Uniprot but with the incorporation of the DFG-out loop conformation from human JNK2. That was necessary to allow the molecular *in silico* docking studies using type II kinase inhibitors, such as compounds A04 and B08. As expected, the inactive state of SmJNK assumed a structural conformation similar to the one observed in the inactive JNK2 protein. Both differ structurally from the active SmJNK as observed in the position of key domains such as the DFG motif, the αC-helix, and the activation loop, which undergoes a structural rearrangement once in its phosphorylated state in active kinases (**Figure 7A**).

Next, control analysis was carried out to select an appropriate *in silico* docking tool from those successfully used before (Beutler et al., 2023). The ligand BIRB796 was separated from the protein chains in the human JNK2 crystal structure coordinate file and re-docked. SwissDock (Grosdidier et al., 2011) as well as the complementary Autodock Vina-based Achilles docking pipeline (Sánchez-Linares et al., 2012) were able to fully re-construct the complex *in silico* exactly as found in the crystal structure. Both approaches were therefore considered reliable and used for subsequent analyses. In contrast to standard docking approaches, we did not impose any assumptions regarding the expected binding site of A04 and B08, e.g. a computational restriction solely to examine the ATP binding pocket (of any of the homology models). Instead, we employed fully blind docking throughout this study. This way, we identified multiple binding sites predicted to be occupied to varying extent by several different, partly overlapping conformeric states of the ligands within both the inactive and active states of SmJNK (data not shown). Both compounds, A04 and B08, exhibited highest affinity to the pocket cavity formed in the inactive SmJNK through multiple multi-modal interactions with several flanking amino acid residues and a total binding affinity of −9.97 kcal/mol and −9.85 kcal/mol, respectively (**Figures 7B, C**; **Supplementary Figure S9A**). Besides, the affinity to the DFG-out conformation is significantly higher compared to DFG-in conformation in the SmJNK AlphaFold2 model, which scored −7.58 kcal/mol and −8.07 kcal/mol for A04 and B08, respectively (**Supplementary Figure S9B**).

Looking closely at the interactions between the ligands and the target SmJNK in its inactive conformation, both compounds were able to form polar interactions with key residues of the cavity pocket. Compounds A04 and B08 formed hydrogen bonds with the conserved glutamate (63E) of the αC-helix, and with the conserved asparagine (D159) of the DFG motif, while compound B08 formed an additional hydrogen bond with a threonine (T173) from the activation loop (**Figures 7B, C**; **Supplementary Figure S9C, D**). Additionally, both compounds showed several hydrophobic interactions with several KLIFS residues, indicating an appropriate docking of the compounds in the ATP pocket of inactive SmJNK. Lastly, in both cases, an aromatic ring of the compound presented aromatic face-to-face interaction with the phenylalanine (F160) of the DFG motif (**Figures 7D, E**).

Although this study cannot rule out the possibility of off-target inhibitory effects, our results clearly suggest preferential binding of the type II inhibitors A04 and B08 to the ATP pocket of SmJNK DFG-out over the DFG-in conformation. These observations reinforce that these compounds can act as type II inhibitors against the JNK protein kinase.

## Discussion

In the present study, we screened 85 small-molecule inhibitors aiming to find novel and promising compounds with high potency against *S. mansoni*. The compounds were *in silico* predicted to target human JNK2 protein kinase by Atomwise, which used its deep learning and artificial intelligence technology for structure-based drug design supported by a library of several million compounds. During the ADMET analysis, four active compounds presented undesirable features, either not complying with the Lipinski’s Rule of Five or being predicted as a promiscuous molecule. Such compounds would not be prioritized in future studies. In contrast, five (A04, A09, A11, B08, and G06) of our priority compounds comply with Lipinski’s Rule of Five for oral absorption, making those attractive starting points for further medicinal chemistry optimization.

The MAP kinase c-Jun N-terminal kinase (JNK) is a well-characterized stress-activated PK and an important mediator of intracellular signaling in mammalian cells in response to extracellular stimuli. Additionally, this protein regulates apoptosis and programmed cell death, which makes it a valuable therapeutic target in cancer research (Wu et al., 2019). In *S. mansoni*, it has been demonstrated that JNK participates as a regulator of parasite maturation, reproduction, and survival (Andrade et al., 2014). Of note, 14 compounds almost completely caused the separation of worm pairs, which is noteworthy considering that female sexual maturation and reproduction are pairing-dependently regulated. This pairing effect has direct consequences for egg production, which is the main pathogenic aspect of schistosomiasis (McManus et al., 2018). A potential role of SmJNK in reproduction was also suggested by RNA-seq studies of adults and their gonads, which showed high SmJNK transcript levels in the testes and especially in the ovaries of paired worms (Lu et al., 2016 and **Supplementary Figure S10**). Following SmJNK RNAi, ovaries of paired females harbored mostly undifferentiated oocytes (Andrade et al., 2014), which suggests roles of SmJNK, as well as other PKs, in the gonad differentiation process especially in females, as hypothesized earlier (Grevelding et al., 2018). Previous research has demonstrated a crosstalk between the SmJNK and Smp38 MAPK signaling pathways, with several genes commonly regulated by both pathways, when these genes were knocked-down in schistosomula. Notably, this regulation included an enrichment of genes with orthologous in *C. elegans* that were associated with RNAi phenotypes leading to nematode sterility (Gava et al., 2019).

Sex and stage specificity are important aspects in drug screening studies. For instance, PZQ can affect adult males or females and juvenile stages of the parasite differently (Gönnert and Andrews, 1977; Pica-Mattoccia and Cioli, 2004). Within the compounds prioritized by Atomwise, we identified a large number of female-specific molecules, i.e. compounds that only presented activity against adult female worms. This was also observed in our previous study, in which we searched for type I kinase inhibitors against SmJNK (Moreira et al., 2022). It is known that female worms have thinner tegumental and subtegumental layers that could facilitate the permeation and effect of drugs. Just like observed in our previous study, our data here suggest that females are more susceptible than males for prioritized inhibitors targeting the JNK protein. Another explanation for this finding is that females express SmJNK in a higher level in the tegument than males. Indeed, cell atlas data show differences in the transcript levels of SmJNK in tegumental cells with a clear bias towards females (Wendt et al., 2020). Furthermore, the effects and phenotypic alterations in some reproductive organs, like those observed for compound A04, suggest that SmJNK inhibitors may affect female worms through mechanisms involved with reproduction. Our data showed that compound B08 was also able to impair cell proliferation in the male and female worms. Thus, compounds that directly affect the stem cell development of this parasite could potentially be explored as alternative therapeutic drugs (Wendt and Collins, 2016). Moreover, SmJNK RNAi knockdown in schistosomula significantly decreased adult worm recovery in a mouse model of infection, which showed considerable morphological alterations, especially in the tegument, in which tubercles were reduced and unusual dilations were observed (Andrade et al., 2014). This finding is supported by additional data on transcript profiles of *S. mansoni* genes during its life cycle (Lu et al., 2018). According to this analysis, SmJNK expression starts early at the schistosomulum stage, increases within the first day of final host infection, and is maintained during the adult stage at nearly equal levels in both genders (**Supplementary Figure S10**). Previous work showed the dependence of tegument integrity from specific somatic stem cells called neoblasts (Wendt et al., 2018). Corresponding to the tegument phenotype we observed, the first single-cell atlas of *S. mansoni* exhibited SmJNK expression in many different cell types including the neoblasts and a neoblast subpopulation leading to tegument progenitor cells, and finally to the tegument (Wendt et al., 2020, **Supplementary Figure S10**). Hence, due to the important roles of JNK in *S. mansoni*, which includes the tegument as a worm coat but also the main surface area for host-parasite interaction, we consider this PK as a highly promising therapeutic target and used it for searching inhibitor compounds.

Gleevec (Imatinib) was the first reported example of a kinase inhibitor that binds to an inactive form of a kinase (Schindler et al., 2000). The authors demonstrated that the activation loop of ABL kinase, which has a different conformation from those of active protein kinases, was not phosphorylated. With this finding, it became possible to achieve high affinity and high specificity when using compounds to exploit the distinctive inactivation mechanisms of each protein kinase. One key feature for this is that amino acid residues surrounding the “type II-pocket” are less conserved as compared to those of type I. Hence, the former can achieve higher levels of selectivity than the latter (Davis et al., 2011). Accordingly, several new type II kinase inhibitors with great potency and selectivity have been identified (Pargellis et al., 2002). To date, 80 kinase inhibitors have been approved by the FDA for therapeutic use in humans, however, none of those specifically targeted JNK, although some drugs do target other members of the MAPK pathway (Roskoski, 2024). To this end, the human JNK2 complexed with the p38α inhibitor BIRB796 was used as a reference structure for the *in silico* screening of type II kinase inhibitors and docking analysis. BIRB796 was the first JNK subfamily-specific DFG-out inhibitor (Kuglstatter et al., 2010), which bound with high affinity to JNK2 (K_d_ = 4.6 nM) (Gruenbaum et al., 2009), lower affinity to JNK3 (K_d_ = 62 nM), but not to JNK1 (Fabian et al., 2005). The co-crystal JNK2-BIRB796 showed that in a DFG-out conformation, the entire activation loop, and consequently the DFG motif, swings out and forms an extended pocket adjacent to the αC-helix, where the compound partially docks (Kuglstatter et al., 2010). This positioning is responsible for blocking JNK2 activation by stabilizing the activation loop in such a way that prevents phosphorylation by upstream kinases. Hence, we hypothesize that the active compounds found here were able to affect the parasite by binding to SmJNK in the same manner. Based on our docking analysis, compounds A04 and B08 form key interactions within inactive SmJNK to several residues that are part of the KLIFS database. KLIFS residues are considered conserved hot spots that accommodate the large diversity of chemical scaffolds in kinase ligands and facilitate the comparative analysis among protein kinases (Van Linden et al., 2014). According to the KLIFS classification, type II inhibitors occupy the front and back clefts, and the gate area of the catalytic cleft. That is the case for both inhibitors, A04 and B08, which present hydrophobic interactions with residues from the G-rich loop, gatekeeper, hinge, and DFG motif (front cleft), but also polar interactions to the back cleft, in this case to the conserved glutamate (E) of the αC-helix. Additionally, the molecular docking analysis showed in both cases hydrogen bonds forming between the ligand and the asparagine (D) of the DFG motif. Stabilization of the DFG motif in specific conformations has already been used as a successful kinase inhibitor strategy (Jeffrey and Robert, 2007; Liao, 2007). Moreover, crystallographic data analysis would help further reveal the details of such interaction.

Although we aim to identify type II inhibitors that target SmJNK, we cannot rule out, at this stage at least, the possibility that the selected active compounds exert off-target effects in our *in vitro* assays. We envision to perform further medicinal chemistry optimization on the best hits and to tackle the issue of selectivity using enzymatic inhibition assays using both parasitic and human homologous recombinant kinases. Notwithstanding, the present work provides an excellent starting point for the identification and further development of novel compounds with high(er) selectivity for the parasite kinases. While crystal structures are available for many human PKs (3700 structures in Uniprot that contain a kinase domain), we are yet to obtain crystal structures for the Schistosoma orthologues, so future studies don’t rely solely in homology models. Recent advances in protein structure prediction has put the study of obscure protein targets in the spotlight, especially after development of prediction algorithms such as AlphaFold (Jumper et al., 2021). However, even the best structural models have their limitations, in particular when comparing conserved proteins or domains, such as the highly conserved ATP binding sites in the human and schistosome PK orthologues. Having such experimental structures will allow us to exploit subtle structural differences within the ATP binding sites, which may or may not be adequately reflected in the structural models. We absolutely do not rule out the possibility that the selected ligands, even after the filtering criteria applied, could also bind to the human PKs or off-target. Nonetheless, this seems to be an intrinsic feature of most drugs that target PKs (Klaeger et al., 2017; Lin et al., 2019).

## Conclusion

In summary, this study strengthens the concept of using MAP kinases as promising drug targets against schistosomiasis. We have shown that the ATP binding pocket of MAP kinases can be further investigated to explore different types of kinase inhibitors other than kinase type I inhibitors. Furthermore, expanding the available chemical space for the search for new SmJNK-specific type II inhibitors might speed up the drug discovery against schistosomiasis. In total, we have identified 33 compounds that were active in at least one of the *in vitro* assays, and one compound presented strong effects against juvenile and adult life stages of *S. mansoni* and good potency against *S. haematobium* adult worms. As it is the case for schistosomes and other human parasites, applying methodologies that reinforce the search for drugs that tackle both stages of the parasite that occur in the human host can be quite beneficial for finding new and more efficient therapeutic treatments for the disease. Moreover, the strategy to screen type II kinase inhibitors resulted in the identification of two compounds that will be further explored.

## Data availability statement

The original contributions presented in the study are included in the article/**Supplementary Material**. Further inquiries can be directed to the corresponding authors.

## Ethics statement

The animal study was approved by Regional Council (Regierungspräsidium) Giessen (V54-19 c 20/15 h 02 GI 18/10 Nr. A 26/2018) and by the Ethics Committee for Animal Use (CEUA) of the Oswaldo Cruz Foundation under license numbers LW-2/22. The study was conducted in accordance with the local legislation and institutional requirements.

## Author contributions

BM: Conceptualization, Data curation, Formal analysis, Investigation, Methodology, Visualization, Writing – original draft, Writing – review & editing. SH: Investigation, Methodology, Visualization, Writing – review & editing, Resources. SGG: Formal analysis, Investigation, Visualization, Writing – review & editing. SG: Investigation, Writing – review & editing. ES: Investigation, Writing – review & editing. MW: Investigation, Resources, Software, Visualization, Writing – review & editing, Conceptualization, Formal analysis. TA: Formal analysis, Investigation, Software, Writing – review & editing. CG: Conceptualization, Data curation, Resources, Writing – review & editing. MM: Conceptualization, Data curation, Formal analysis, Methodology, Project administration, Supervision, Writing – review & editing, Funding acquisition. FF: Conceptualization, Funding acquisition, Project administration, Supervision, Writing – review & editing, Resources.

## References

[B1] Aboagye I. F. Addison Y. A. A. (2023). Praziquantel efficacy, urinary and intestinal schistosomiasis reinfection–a systematic review. Pathog. Glob Health 117, 623–630. doi: 10.1080/20477724.2022.2145070 36394218 PMC10498796

[B2] Adasme M. F. Linnemann K. L. Bolz S. N. Kaiser F. Salentin S. Haupt V. J.. (2021). PLIP 2021: Expanding the scope of the protein-ligand interaction profiler to DNA and RNA. Nucleic Acids Res. 49, W530–W534. doi: 10.1093/nar/gkab294 33950214 PMC8262720

[B3] Aguiar P. H. N. Fernandes N. M. G. S. Zani C. L. Mourão M. M. (2017). A high-throughput colorimetric assay for detection of *Schistosoma mansoni* viability based on the tetrazolium salt XTT. Parasit Vectors 10, 300. doi: 10.1186/s13071-017-2240-3 28637488 PMC5480175

[B4] Andrade L. F. de Mourão M. M. Geraldo J. A. Coelho F. S. Silva L. L. Neves R. H.. (2014). Regulation of *schistosoma mansoni* development and reproduction by the mitogen-activated protein kinase signaling pathway. PloS Negl. Trop. Dis. 8, e3081. doi: 10.1371/journal.pntd.0002949 PMC4063740 24945272

[B5] Andrade L. F. Nahum L. A. Avelar L. G. A. Silva L. L. Zerlotini A. Ruiz J. C.. (2011). Eukaryotic protein kinases (ePKs) of the helminth parasite *schistosoma mansoni* . BMC Genomics 12, 215. doi: 10.1186/1471-2164-12-215 21548963 PMC3117856

[B6] Arab S. S. Dantism A. (2023). EasyModel: a user-friendly web-based interface based on MODELLER. Sci. Rep. 13, 17185. doi: 10.1038/s41598-023-44505-9 37821634 PMC10567746

[B7] Avelar L. das G. A. Gava S. G. Neves R. H. Soares Silva M. C. Araújo N.. (2019). Smp38 MAP kinase regulation in *Schistosoma mansoni*: Roles in survival, oviposition, and protection against oxidative stress. Front. Immunol. 10. doi: 10.3389/fimmu.2019.00021 PMC6353789 30733716

[B8] Baell J. B. Holloway G. A. (2010). New substructure filters for removal of pan assay interference compounds (PAINS) from screening libraries and for their exclusion in bioassays. J. Med. Chem. 53, 2719–2740. doi: 10.1021/jm901137j 20131845

[B9] Beckmann S. (2012). Protein kinases as potential targets for novel anti-schistosomal strategies. Curr. Pharm. Des. 18, 3579–3594. doi: 10.2174/138161212801327310 22607148

[B10] Beckmann S. Grevelding C. G. (2010). Imatinib has a fatal impact on morphology, pairing stability and survival of adult *Schistosoma mansoni in vitro* . Int. J. Parasitol. 40, 521–526. doi: 10.1016/j.ijpara.2010.01.007 20149792

[B11] Beutler M. Harnischfeger J. Weber M. H. W. Hahnel S. R. Quack T. Blohm A.. (2023). Identification and characterisation of the tegument-expressed aldehyde dehydrogenase SmALDH_312 of *Schistosoma mansoni*, a target of disulfiram. Eur. J. Med. Chem. 251, 115179. doi: 10.1016/j.ejmech.2023.115179 36948075

[B12] Bruns R. F. Watson I. A. (2012). Rules for identifying potentially reactive or promiscuous compounds. J. Med. Chem. 55, 9763–9772. doi: 10.1021/jm301008n 23061697

[B13] Burke M. L. Jones M. K. Gobert G. N. Li Y. S. Ellis M. K. McManus D. P. (2009). Immunopathogenesis of human schistosomiasis. Parasite Immunol. 31, 163–176. doi: 10.1111/j.1365-3024.2009.01098.x 19292768

[B14] Butina D. (1999). Unsupervised data base clustering based on daylight’s fingerprint and Tanimoto similarity: A fast and automated way to cluster small and large data sets. J. Chem. Inf Comput. Sci. 39, 747–750. doi: 10.1021/ci9803381

[B15] Caldwell N. Afshar R. Baragaña B. Bustinduy A. L. Caffrey C. R. Collins J. J.. (2023). Perspective on schistosomiasis drug discovery: highlights from a schistosomiasis drug discovery workshop at wellcome collection, london, september 2022. ACS Infect. Dis. 9, 1046–1055. doi: 10.1021/acsinfecdis.3c00081 37083395 PMC10186373

[B16] Cheever A. W. Macedonia J. G. Mosimann J. E. Cheever E. A. (1994). Kinetics of egg production and egg excretion by *Schistosoma mansoni* and *S. japonicum* in mice infected with a single pair of worms. Am. J. Trop. Med. Hygiene 50, 281–295. doi: 10.4269/ajtmh.1994.50.281 8147487

[B17] Colley D. G. Bustinduy A. L. Secor W. E. King C. H. (2014). Human schistosomiasis. Lancet 383, 2253–2264. doi: 10.1016/S0140-6736(13)61949-2 24698483 PMC4672382

[B18] Daina A. Michielin O. Zoete V. (2017). SwissADME: A free web tool to evaluate pharmacokinetics, drug-likeness and medicinal chemistry friendliness of small molecules. Sci. Rep. 7, 42717. doi: 10.1038/srep42717 28256516 PMC5335600

[B19] Dar A. C. Shokat K. M. (2011). The evolution of protein kinase inhibitors from antagonists to agonists of cellular signaling. Annu. Rev. Biochem. 80, 769–795. doi: 10.1146/annurev-biochem-090308-173656 21548788

[B20] Davis M. I. Hunt J. P. Herrgard S. Ciceri P. Wodicka L. M. Pallares G.. (2011). Comprehensive analysis of kinase inhibitor selectivity. Nat. Biotechnol. 29, 1046–1051. doi: 10.1038/nbt.1990 22037378

[B21] Delano W. L. (2002). The pyMOL molecular graphics system. CCP4 Newslett. Protein Crystallogr. 40.

[B22] Dettman C. D. Higgins-Opitz S. B. Saikoolal A. (1989). Enhanced efficacy of the paddling method for schistosome infection of rodents by a four-step pre-soaking procedure. Parasitol. Res. 76, 183–184. doi: 10.1007/BF00930846 2515540

[B23] Fabian M. A. Biggs W. H. Treiber D. K. Atteridge C. E. Azimioara M. D. Benedetti M. G.. (2005). A small molecule-kinase interaction map for clinical kinase inhibitors. Nat. Biotechnol. 23, 329–336. doi: 10.1038/nbt1068 15711537

[B24] Gava S. G. Tavares N. C. Falcone F. H. Oliveira G. Mourão M. M. (2019). Profiling Transcriptional Regulation and Functional Roles of *Schistosoma mansoni* c-Jun N-Terminal Kinase. Front. Genet. 10. doi: 10.3389/fgene.2019.01036 PMC6813216 31681440

[B25] Gelmedin V. Dissous C. Grevelding C. G. (2015). Re-positioning protein-kinase inhibitors against schistosomiasis. Future Med. Chem. 7, 737–752. doi: 10.4155/fmc.15.31 25996067

[B26] Gönnert R. Andrews P. (1977). Praziquantel, a new broad-spectrum antischistosomal agent. Z. für Parasitenkunde 52, 129–150. doi: 10.1007/BF00389899 410178

[B27] Grevelding C. G. Langner S. Dissous C. (2018). Kinases: molecular stage directors for schistosome development and differentiation. Trends Parasitol. 34, 246–260. doi: 10.1016/j.pt.2017.12.001 29276074

[B28] Grevelding C. G. Sommer G. Kunz W. (1997). Female-specific gene expression in *Schistosoma mansoni* is regulated by pairing. Parasitology 115, 635–640. doi: 10.1017/S0031182097001728 9488875

[B29] Grosdidier A. Zoete V. Michielin O. (2011). SwissDock, a protein-small molecule docking web service based on EADock DSS. Nucleic Acids Res. 39, W270–W277. doi: 10.1093/nar/gkr366 21624888 PMC3125772

[B30] Gruenbaum L. M. Schwartz R. Woska J. R. DeLeon R. P. Peet G. W. Warren T. C.. (2009). Inhibition of pro-inflammatory cytokine production by the dual p38/JNK2 inhibitor BIRB796 correlates with the inhibition of p38 signaling. Biochem. Pharmacol. 77, 422–432. doi: 10.1016/j.bcp.2008.10.032 19027720

[B31] Gu Z. Eils R. Schlesner M. (2016). Complex heatmaps reveal patterns and correlations in multidimensional genomic data. Bioinformatics 32, 2847–2849. doi: 10.1093/bioinformatics/btw313 27207943

[B32] Jeffrey L. Robert A. (2007). Targeting protein multiple conformations: A structure-based strategy for kinase drug design. Curr. Top. Med. Chem. 7, 1394–1407. doi: 10.2174/156802607781696783 17692028

[B33] Jumper J. Evans R. Pritzel A. Green T. Figurnov M. Ronneberger O.. (2021). Highly accurate protein structure prediction with AlphaFold. Nature 596, 583–589. doi: 10.1038/s41586-021-03819-2 34265844 PMC8371605

[B34] Kabuyaya M. Chimbari M. J. Mukaratirwa S. (2018). Efficacy of praziquantel treatment regimens in pre-school and school aged children infected with schistosomiasis in sub-Saharan Africa: A systematic review. Infect. Dis. Poverty 7. doi: 10.1186/s40249-018-0448-x PMC6036702 29986763

[B35] Kellershohn J. Thomas L. Hahnel S. R. Grünweller A. Hartmann R. K. Hardt M.. (2018). Insects in anthelminthics research: Lady beetle-derived harmonine affects survival, reproduction and stem cell proliferation of *Schistosoma mansoni* . PloS Negl. Trop. Dis. 13, e0007240. doi: 10.1371/journal.pntd.0007240 PMC6436750 30870428

[B36] Kelley L. A. Mezulis S. Yates C. M. Wass M. N. Sternberg M. J. E. (2015). The Phyre2 web portal for protein modeling, prediction and analysis. Nat. Protoc. 10, 845–858. doi: 10.1038/nprot.2015.053 25950237 PMC5298202

[B37] Klaeger S. Heinzlmeir S. Wilhelm M. Polzer H. Vick B. Koenig P. A.. (2017). The target landscape of clinical kinase drugs. Sci. (1979) 358. doi: 10.1126/science.aan4368 PMC6542668 29191878

[B38] Kuglstatter A. Ghate M. Tsing S. Villaseñor A. G. Shaw D. Barnett J. W.. (2010). X-ray crystal structure of JNK2 complexed with the p38α inhibitor BIRB796: Insights into the rational design of DFG-out binding MAP kinase inhibitors. Bioorg Med. Chem. Lett. 20, 5217–5220. doi: 10.1016/j.bmcl.2010.06.157 20655210

[B39] Kunz W. (2001). Schistosome male-female interaction: induction of germ-cell differentiation. Trends Parasitol. 17, 227–231. doi: 10.1016/S1471-4922(01)01893-1 11323306

[B40] Le Clec’h W. Chevalier F. D. Mattos A. C. A. Strickland A. Diaz R. McDew-White M.. (2021). Genetic analysis of praziquantel response in schistosome parasites implicates a transient receptor potential channel. Sci. Transl. Med. 13. doi: 10.1126/scitranslmed.abj9114 PMC9491494 34936381

[B41] Li X. Haeberlein S. Zhao L. Mughal M. N. Zhu T. Liu L.. (2019). The ABL kinase inhibitor imatinib causes phenotypic changes and lethality in adult *Schistosoma japonicum* . Parasitol. Res. 118, 881–890. doi: 10.1007/s00436-019-06224-x 30729300

[B42] Liao J.J.L. (2007). Molecular recognition of protein kinase binding pockets for design of potent and selective kinase inhibitors. J. Med. Chem. 50, 409–424. doi: 10.1021/jm0608107 17266192

[B43] Lin A. Giuliano C. J. Palladino A. John K. M. Abramowicz C. Yuan M.L.. (2019). Off-target toxicity is a common mechanism of action of cancer drugs undergoing clinical trials. Sci. Transl. Med. 11. doi: 10.1126/scitranslmed.aaw8412 PMC7717492 31511426

[B44] LoVerde P. T. (2019). “Schistosomiasis,” in Digenetic Treamatodes, 45–70. doi: 10.1007/978-3-030-18616-6_3 31297759

[B45] LoVerde P. T. Andrade L. F. Oliveira G. (2009). Signal transduction regulates schistosome reproductive biology. Curr. Opin. Microbiol. 12, 422–428. doi: 10.1016/j.mib.2009.06.005 19577949 PMC2740793

[B46] Lu Z. Sessler F. Holroyd N. Hahnel S. Quack T. Berriman M.. (2016). Schistosome sex matters: A deep view into gonad-specific and pairing-dependent transcriptomes reveals a complex gender interplay. Sci. Rep. 6, 1–14. doi: 10.1038/srep31150 27499125 PMC4976352

[B47] Lu Z. Zhang Y. Berriman M. (2018). A web portal for gene expression across all life stages of *Schistosoma mansoni* . bioRxiv. doi: 10.1101/308213

[B48] Mäder P. Rennar G. A. Ventura A. M. P. Grevelding C. G. Schlitzer M. (2018). Chemotherapy for fighting schistosomiasis: past, present and future. ChemMedChem 13, 2374–2389. doi: 10.1002/cmdc.201800572 30212614

[B49] Marcellino C. Gut J. Lim K. C. Singh R. McKerrow J. Sakanari J. (2012). WormAssay: A novel computer application for whole-plate motion-based screening of macroscopic parasites. PloS Negl. Trop. Dis. 6, e1494. doi: 10.1371/journal.pntd.0001494 22303493 PMC3269415

[B50] McManus D. P. Dunne D. W. Sacko M. Utzinger J. Vennervald B. J. Zhou X. N. (2018). Schistosomiasis. Nat. Rev. Dis. Primers 4, 13. doi: 10.1038/s41572-018-0013-8 30093684

[B51] Milligan J. N. Jolly E. R. (2011). Cercarial Transformation and in *vitro* Cultivation of *Schistosoma mansoni* Schistosomules. J. Visualized Experiments 54, E3191. doi: 10.3791/3191-v PMC3217644 21876520

[B52] Mirdita M. Schütze K. Moriwaki Y. Heo L. Ovchinnikov S. Steinegger M. (2022). ColabFold: making protein folding accessible to all. Nat. Methods 19, 679–682. doi: 10.1038/s41592-022-01488-1 35637307 PMC9184281

[B53] Moreira B. P. Batista I. C. A. Tavares N. C. Armstrong T. Gava S. G. Torres G. P.. (2022). Docking-based virtual screening enables prioritizing protein kinase inhibitors with *in vitro* phenotypic activity against *schistosoma mansoni* . Front. Cell Infect. Microbiol. 12. doi: 10.3389/fcimb.2022.913301 PMC9294739 35865824

[B54] Mulnaes D. Koenig F. Gohlke H. (2021). TopSuite web server: A meta-suite for deep-learning-based protein structure and quality prediction. J. Chem. Inf Model. 61, 548–553. doi: 10.1021/acs.jcim.0c01202 33464891

[B55] Mulnaes D. Porta N. Clemens R. Apanasenko I. Reiners J. Gremer L.. (2020). TopModel: template-based protein structure prediction at low sequence identity using top-down consensus and deep neural networks. J. Chem. Theory Comput. 16, 1953–1967. doi: 10.1021/acs.jctc.9b00825 31967823

[B56] Nogueira R. A. Lira M. G. S. Licá I. C. L. Frazão G. C. C. G. dos Santos V. A. F. Filho A. C. C. M.. (2022). Praziquantel: An update on the mechanism of its action against schistosomiasis and new therapeutic perspectives. Mol. Biochem. Parasitol. 252, 111531. doi: 10.1016/j.molbiopara.2022.111531 36375598

[B57] Pargellis C. Tong L. Churchill L. Cirillo P. F. Gilmore T. Graham A. G.. (2002). Inhibition of p38 MAP kinase by utilizing a novel allosteric binding site. Nat. Struct. Biol. 9, 268–272. doi: 10.1038/nsb770 11896401

[B58] Pellegrino J. Siqueira A. (1956). A perfusion technic for recovery of *Schistosoma mansoni* from experimentally infected Guinea pigs. Rev. Bras. Malariologia e Doencas Tropicais. 8, 589–597. 13494879

[B59] Pettersen E. F. Goddard T. D. Huang C. C. Couch G. S. Greenblatt D. M. Meng E. C.. (2004). UCSF Chimera - A visualization system for exploratory research and analysis. J. Comput. Chem. 25, 1605–1612. doi: 10.1002/jcc.20084 15264254

[B60] Pettersen E. F. Goddard T. D. Huang C. C. Meng E. C. Couch G. S. Croll T. I.. (2021). UCSF ChimeraX: Structure visualization for researchers, educators, and developers. Protein Sci. 30, 70–82. doi: 10.1002/pro.3943 32881101 PMC7737788

[B61] Pica-Mattoccia L. Cioli D. (2004). Sex- and stage-related sensitivity of *Schistosoma mansoni* to in *vivo* and in *vitro* praziquantel treatment. Int. J. Parasitol. 34, 527–533. doi: 10.1016/j.ijpara.2003.12.003 15013742

[B62] Pires D. E. V. Blundell T. L. Ascher D. B. (2015). pkCSM: Predicting small-molecule pharmacokinetic and toxicity properties using graph-based signatures. J. Med. Chem. 58, 4066–4072. doi: 10.1021/acs.jmedchem.5b00104 25860834 PMC4434528

[B63] Popiel I. Basch P. F. (1984). Reproductive development of female *Schistosoma mansoni* (Digenea: Schistosomatidae) following bisexual pairing of worms and worm segments. J. Exp. Zool. 232, 141–150. doi: 10.1002/jez.1402320117 6502090

[B64] R Core Team. (2023). R: A language and environment for statistical computing. Vienna, Austria: R Foundation for Statistical Computing. Available at: https://www.R-project.org/.

[B65] Ressurreição M. Rollinson D. Emery A. M. Walker A. J. (2011). A role for p38 mitogen-activated protein kinase in early post-embryonic development of *Schistosoma mansoni* . Mol. Biochem. Parasitol. 180, 51–55. doi: 10.1016/j.molbiopara.2011.07.002 21787807

[B66] Rodgers D. Hahn M. (2010). Extended-connectivity fingerprints. J. Chem. Inf. Model. 50, 742–754. doi: 10.1021/ci100050t 20426451

[B67] Roskoski R. (2024). Properties of FDA-approved small molecule protein kinase inhibitors: A 2024 update. Pharmacol. Res. 200, 107059. doi: 10.1016/j.phrs.2024.107059 38216005

[B68] Roy A. Kucukural A. Zhang Y. (2010). I-TASSER: A unified platform for automated protein structure and function prediction. Nat. Protoc. 5, 725–738. doi: 10.1038/nprot.2010.5 20360767 PMC2849174

[B69] Sánchez-Linares I. Pérez-Sánchez H. Cecilia J. M. García J. M. (2012). High-Throughput parallel blind Virtual Screening using BINDSURF. BMC Bioinf. 13, S13. doi: 10.1186/1471-2105-13-S14-S13 PMC3504923 23095663

[B70] Schindler T. Bornmann W. Pellicena P. Miller W. T. Clarkson B. Kuriyan J. (2000). Structural mechanism for STI-571 inhibition of Abelson tyrosine kinase. Sci. (1979) 289, 1938–1942. doi: 10.1126/science.289.5486.1938 10988075

[B71] Sievers F. Wilm A. Dineen D. Gibson T. J. Karplus K. Li W.. (2011). Fast, scalable generation of high-quality protein multiple sequence alignments using Clustal Omega. Mol. Syst. Biol. 7. doi: 10.1038/msb.2011.75 PMC3261699 21988835

[B72] Song Y. Dimaio F. Wang R. Y. R. Kim D. Miles C. Brunette T.. (2013). High-resolution comparative modeling with RosettaCM. Structure 21, 1735–1742. doi: 10.1016/j.str.2013.08.005 24035711 PMC3811137

[B73] Souza Silva J. A. Tunes L. G. Coimbra R. S. Ascher D. B. Pires D. E. V. Monte-Neto R. L. (2021). Unveiling six potent and highly selective antileishmanial agents via the open source compound collection ‘Pathogen Box’ against antimony-sensitive and -resistant *Leishmania Braziliensis* . Biomed. Pharmacother. 133, 111049. doi: 10.1016/j.biopha.2020.111049 33378956

[B74] Stafford K. A. Anderson B. M. Sorenson J. Van Den Bedem H. (2022). AtomNet poseRanker: enriching ligand pose quality for dynamic proteins in virtual high-throughput screens. J. Chem. Inf Model. 62, 1178–1189. doi: 10.1021/acs.jcim.1c01250 35235748 PMC8924924

[B75] Stroehlein A. J. Young N. D. Jex A. R. Sternberg P. W. Tan P. Boag P. R.. (2015). Defining the *Schistosoma haematobium* kinome enables the prediction of essential kinases as anti-schistosome drug targets. Sci. Rep. 5, 17759. doi: 10.1038/srep17759 26635209 PMC4669435

[B76] Tavares N. C. Mourão M. M. (2021). Parasitemia evaluation in mice infected with schistosoma mansoni. Bio Protoc. 11. doi: 10.21769/BioProtoc.4017 PMC8187114 34150924

[B77] The Atomwise AIMS Program. (2024). AI is a viable alternative to high throughput screening: a 318-target study. Sci. Rep. 14, 7526. doi: 10.1038/s41598-024-54655-z 38565852 PMC10987645

[B78] Valentim C. L. L. Cioli D. Chevalier F. D. Cao X. Taylor A. B. Holloway S. P.. (2013). Genetic and molecular basis of drug resistance and species-specific drug action in Schistosome parasites. Sci. (1979) 342, 1385–1389. doi: 10.1126/science.1243106 PMC4136436 24263136

[B79] Van Linden O. P. J. Kooistra A. J. Leurs R. De Esch I. J. P. De Graaf C. (2014). KLIFS: A knowledge-based structural database to navigate kinase-ligand interaction space. J. Med. Chem. 57, 249–277. doi: 10.1021/jm400378w 23941661

[B80] Varadi M. Anyango S. Deshpande M. Nair S. Natassia C. Yordanova G.. (2022). AlphaFold Protein Structure Database: Massively expanding the structural coverage of protein-sequence space with high-accuracy models. Nucleic Acids Res. 50, D439–D444. doi: 10.1093/nar/gkab1061 34791371 PMC8728224

[B81] Waechtler A. Cezanne B. Maillard D. Sun R. Wang S. Wang J.. (2023). Praziquantel – 50 years of research. ChemMedChem 18. doi: 10.1002/cmdc.202300154 37009677

[B82] Wallach I. Dzamba M. Heifets A. (2015). AtomNet: A deep convolutional neural network for bioactivity prediction in structure-based drug discovery. doi: 10.48550/arXiv.1510.02855

[B83] Wang W. Wang L. Liang Y. S. (2012). Susceptibility or resistance of praziquantel in human schistosomiasis: A review. Parasitol. Res. 111, 1871–1877. doi: 10.1007/s00436-012-3151-z 23052781

[B84] Wang L. Yang Z. Li Y. Yu F. Brindley P. J. McManus D. P.. (2006). Reconstruction and in silico analysis of the MAPK signaling pathways in the human blood fluke, *Schistosoma japonicum* . FEBS Lett. 580, 3677–3686. doi: 10.1016/j.febslet.2006.05.055 16765950

[B85] Waterhouse A. Bertoni M. Bienert S. Studer G. Tauriello G. Gumienny R.. (2018). SWISS-MODEL: Homology modeling of protein structures and complexes. Nucleic Acids Res. 46, W296–W303. doi: 10.1093/nar/gky427 29788355 PMC6030848

[B86] Webb B. Sali A. (2016). Comparative protein structure modeling using MODELLER. Curr. Protoc. Bioinf. 54. doi: 10.1002/cpbi.3 PMC5031415 27322406

[B87] Wendt G. R. Collins J. J. (2016). Schistosomiasis as a disease of stem cells. Curr. Opin. Genet. Dev. 40, 95–102. doi: 10.1016/j.gde.2016.06.010 27392295 PMC5135665

[B88] Wendt G. R. Collins J. N. Pei J. Pearson M. S. Bennett H. M. Loukas A.. (2018). Flatworm-specific transcriptional regulators promote the specification of tegumental progenitors in *Schistosoma mansoni* . Elife 7, 1644–1649. doi: 10.7554/eLife.33221.033 PMC5927768 29557781

[B89] Wendt G. Zhao L. Chen R. Liu C. O’Donoghue A. J. Caffrey C. R.. (2020). A single-cell RNA-seq atlas of *Schistosoma mansoni* identifies a key regulator of blood feeding. Sci. (1979) 369, 1644–1649. doi: 10.1126/science.abb7709 PMC7875187 32973030

[B90] WHO. (2023). World health organization. Available at: https://www.who.int/news-room/fact-sheets/detail/schistosomiasis (Accessed July 5, 2023).

[B91] Wu Q. Wu W. Fu B. Shi L. Wang X. Kuca K. (2019). JNK signaling in cancer cell survival. Med. Res. Rev. 39, 2082–2104. doi: 10.1002/med.21574 30912203

[B92] Wu K. Zhai X. Huang S. Jiang L. Yu Z. Huang J. (2021). Protein kinases: potential drug targets against *schistosoma japonicum* . Front. Cell Infect. Microbiol. 11. doi: 10.3389/fcimb.2021.691757 PMC8282181 34277472

