# Supplementary material for: Identification of potent schistosomicidal compounds predicted as type II-kinase inhibitors against Schistosoma mansoni c-Jun N-terminal kinase SMJNK
Source: Front Parasitol. 2024 Apr 26;3:1394407. doi: 10.3389/fpara.2024.1394407 (PMC11732180; doi:10.3389/fpara.2024.1394407)
Supplement: Supplementary file 1 [file DataSheet_1.docx]

**Supplementary Material**

**Supplementary Table S1.** Characteristics of compounds and predicted ADMET properties. The first sheet lists 85 compounds provided by Atomwise and additional compound characteristics. The second sheet shows the result of the ADMET analysis performed by the pkCSM tool, including molecular weight, logP, number of rotatable bonds, number of hydrogen bond acceptors and donors, surface area, water solubility, Caco-2 permeability, intestinal absorption (human), skin permeability, p-glycoprotein substrate, p-glycoprotein I and II inhibitors, VDSs (human), fraction unbound (human), BBB and CNS permeability, CYP2D6, CYP3A4, CYP1A2, CYP2C19, CYP2C9, CYP2D6, and CYP3A4 substrate, total clearance, renal OCT2 substrate, AMES toxicity, maximum tolerated dose (human), hERG I and II inhibitors, oral rat acute toxicity (LD50), oral rat chronic toxicity (LOAEL), hepatotoxicity, skin sensitisation, *T. pyriformis* toxicity, minnow toxicity and the calculated pkCSM SCORE. The third sheet shows the result of the ADMET analysis performed by the SwissADME tool.

**Supplementary Figures**

**
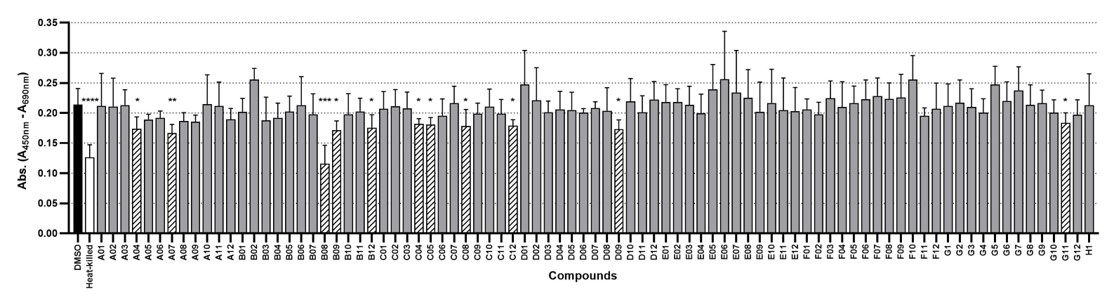
**

**Supplementary Figure S1.** Bar representation of schistosomula viability measured by XTT assay after treatment with 20 µM compound for 48 h. Plotted values (mean + SD) represent biological replicates (n ≥ 3) and non-parametric Kruskal-Wallis test with uncorrected Dunn’s test (p-value < 0.0332 (*); < 0.0021 (**); < 0.0002 (***); < 0.0001(****)) was used for statistical analysis.


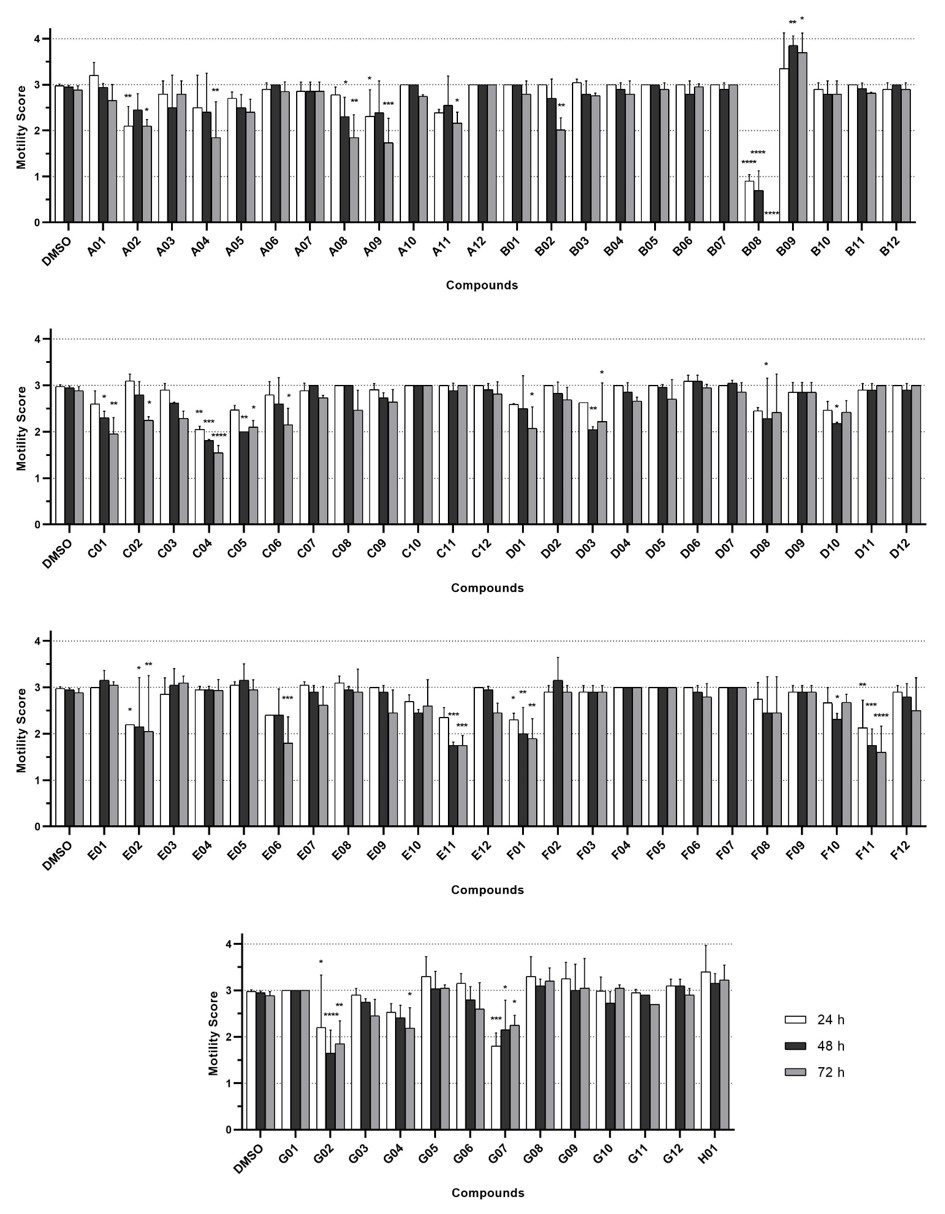


**Supplementary Figure S2.** Bar representation of the motility score of 85 compounds at 24-, 48-, and 72-hour timepoints after compound incubation at 20 µM. Worms were observed daily and a score was given to each worm (or pair of worms) as described in the Methods section. Plotted scores (mean + SD) represent two independent experiments and two-way ANOVA with uncorrected Fisher’s LSD (p-value < 0.0332 (*); < 0.0021 (**); < 0.0002 (***); < 0.0001(****)) was performed.


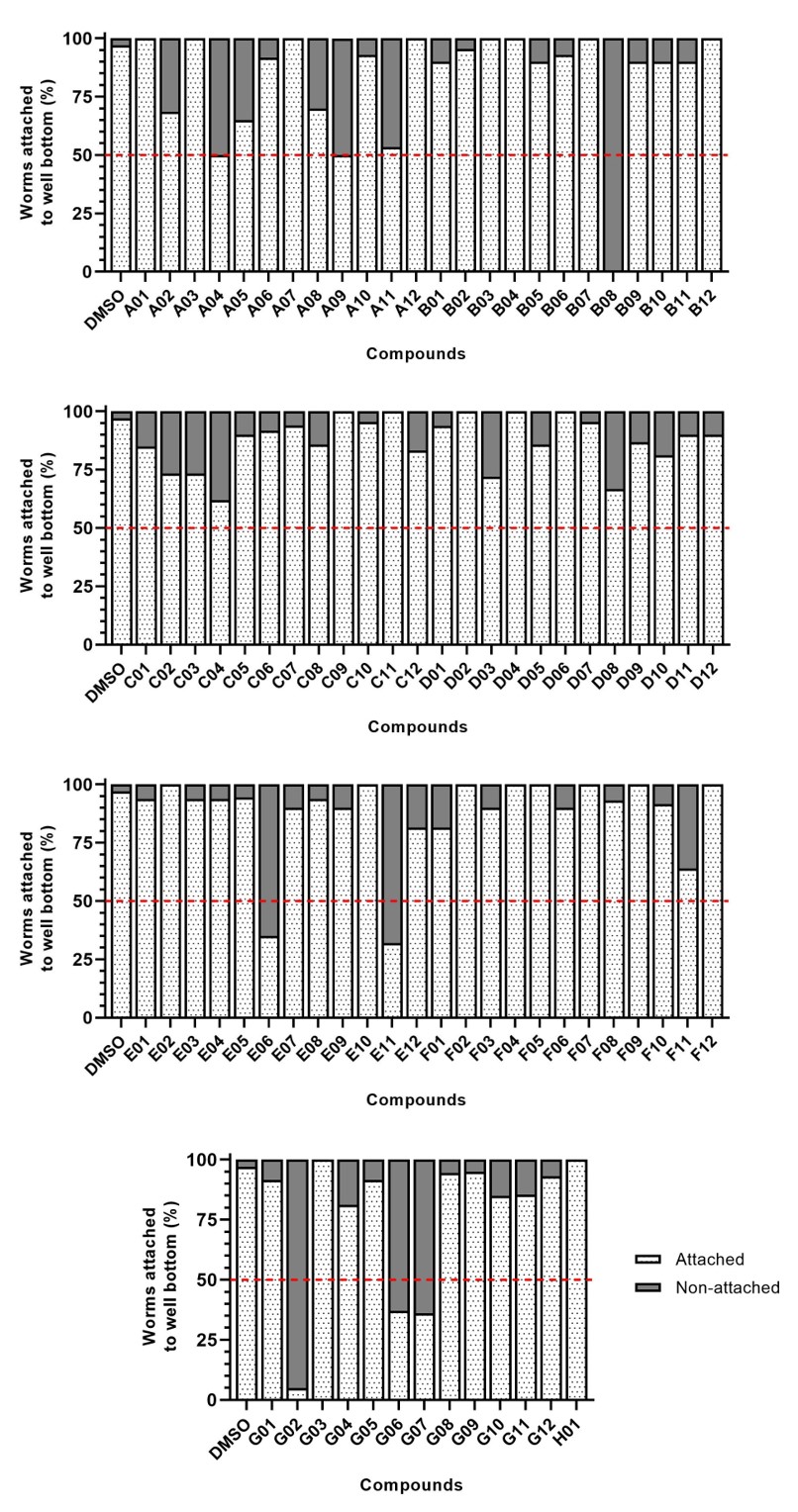


**Supplementary Figure S3.** Bar representation of the percentage of the attachment status of worms after 72 hours of incubation with each of the 85 tested compounds at 20 µM. Plotted scores (mean) represent two independent experiments.


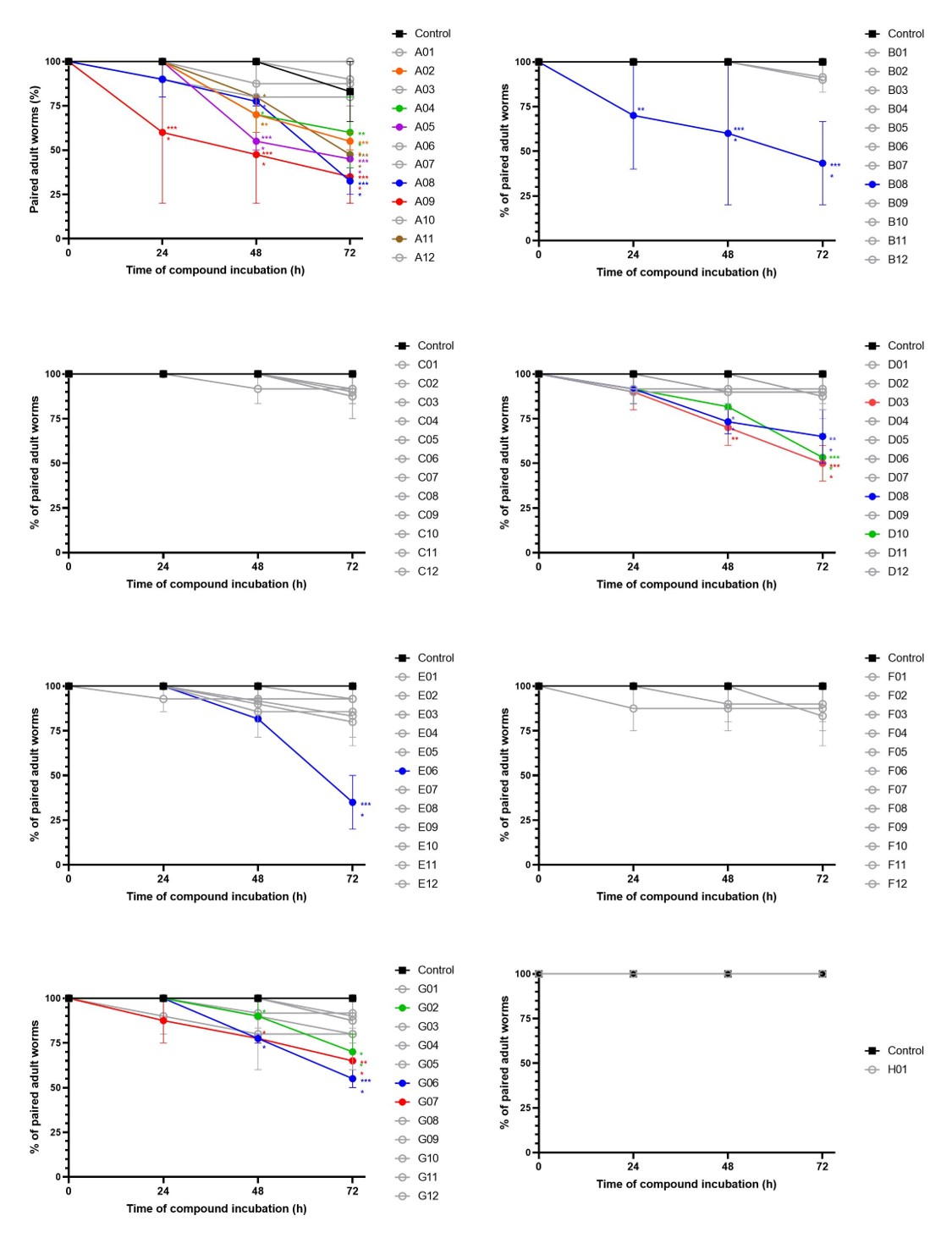


**Supplementary Figure S4.** Points-and-line graph representation of the percentage of paired worms at each observed timepoint after incubation with each of the 85 tested compounds at 20 µM. Plotted scores (mean + SD) represent two independent experiments and two-way ANOVA with uncorrected Fisher’s LSD (p-value < 0.0332 (*); < 0.0021 (**); < 0.0002 (***); < 0.0001(****)) was performed.


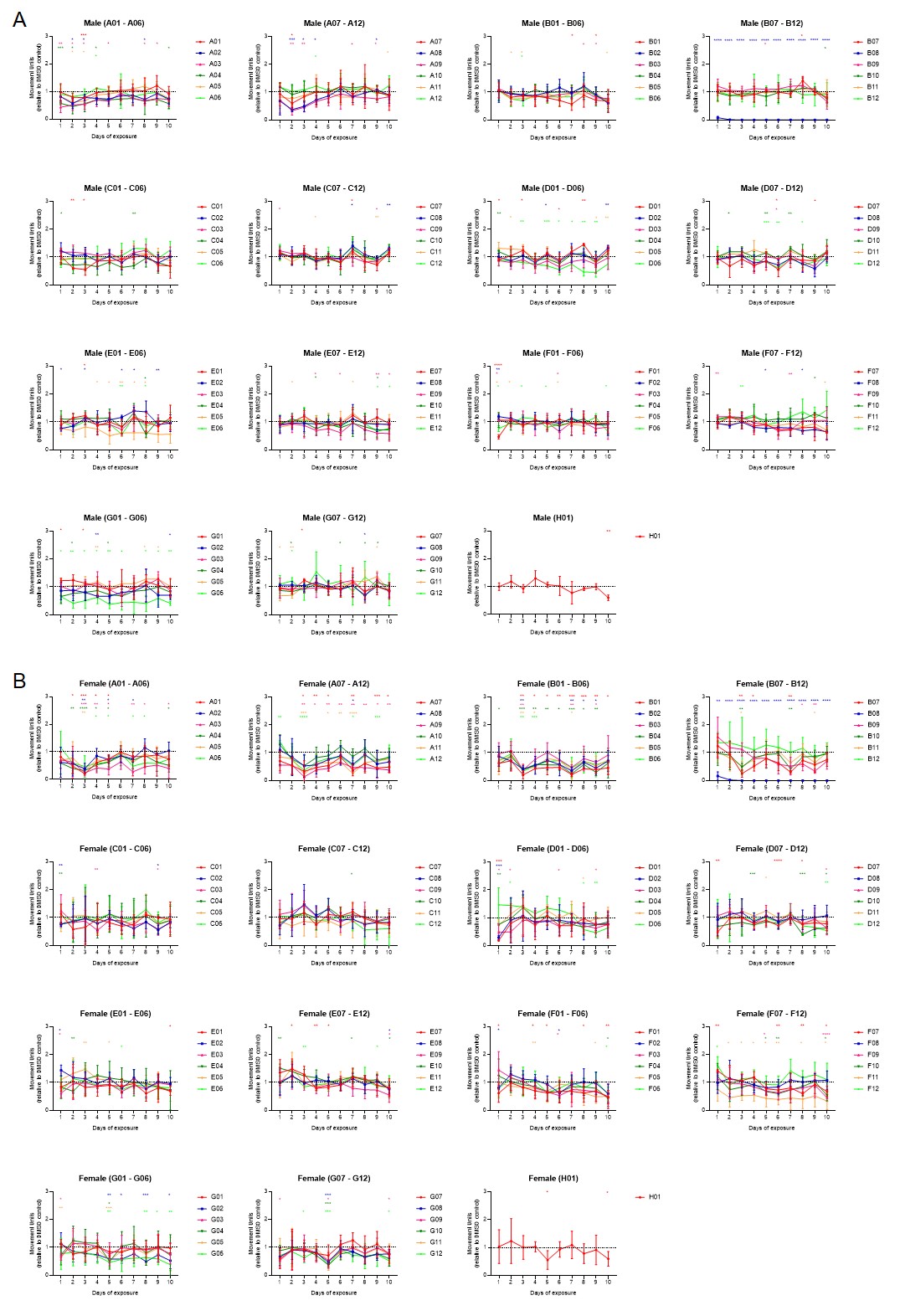


**Supplementary Figure S5.** Effect of compounds on the motility of male and female worms. Points-and-line graph representation of the mean of the movement units of male (A) and female (B) adult worms exposed to compounds at 20 µM over ten days. The movement units of worms exposed to the compounds were normalised relative to the movement units of worms exposed to the vehicle control DMSO 0.4% (red dotted line). Error bars are represented above the plotted data. Statistical analyses using mixed-effects model are represented with asterisks above the points (p-value < 0.05 (*); < 0.01 (**); < 0.001 (***); < 0.0001 (****)).


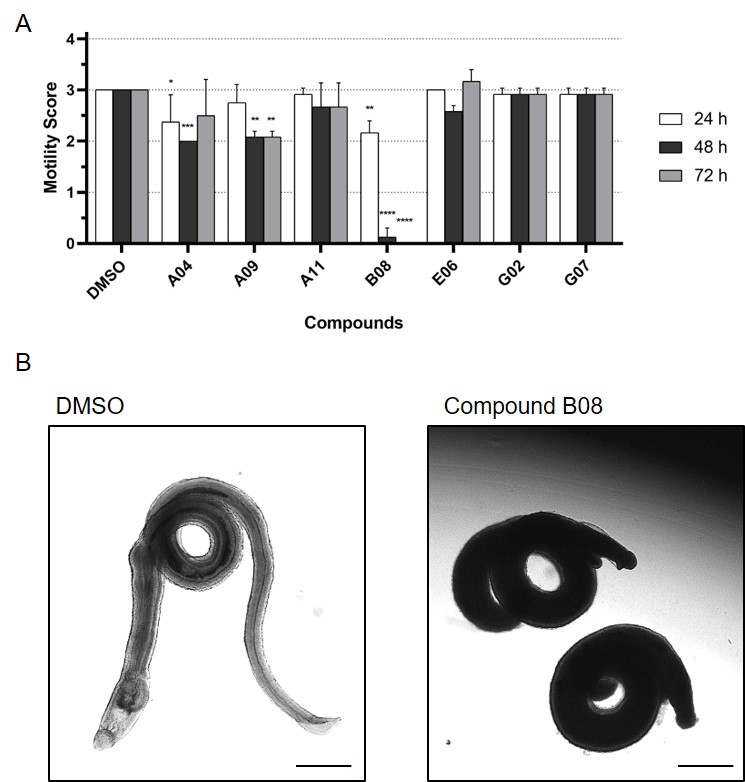


**Supplementary Figure S6.** Compound B08 strongly affects male *S. haematobium* adult worms. A) Bar representation of the motility score of compounds at 24-, 48-, and 72-hour timepoints after compound incubation. Worms were observed daily, and a score was given to each worm as described in the Methods section. Plotted scores (mean + SD) represent two independent experiments and two-way ANOVA with uncorrected Fisher’s LSD (p value < 0.0332 (*); < 0.0021 (**); < 0.0002 (***); < 0.0001(****)) was performed. B) Light microscopy (bright field) of an adult worm (male) after 48 hours of incubation with 20 µM of compound B08. Scale bar: 500 µm.


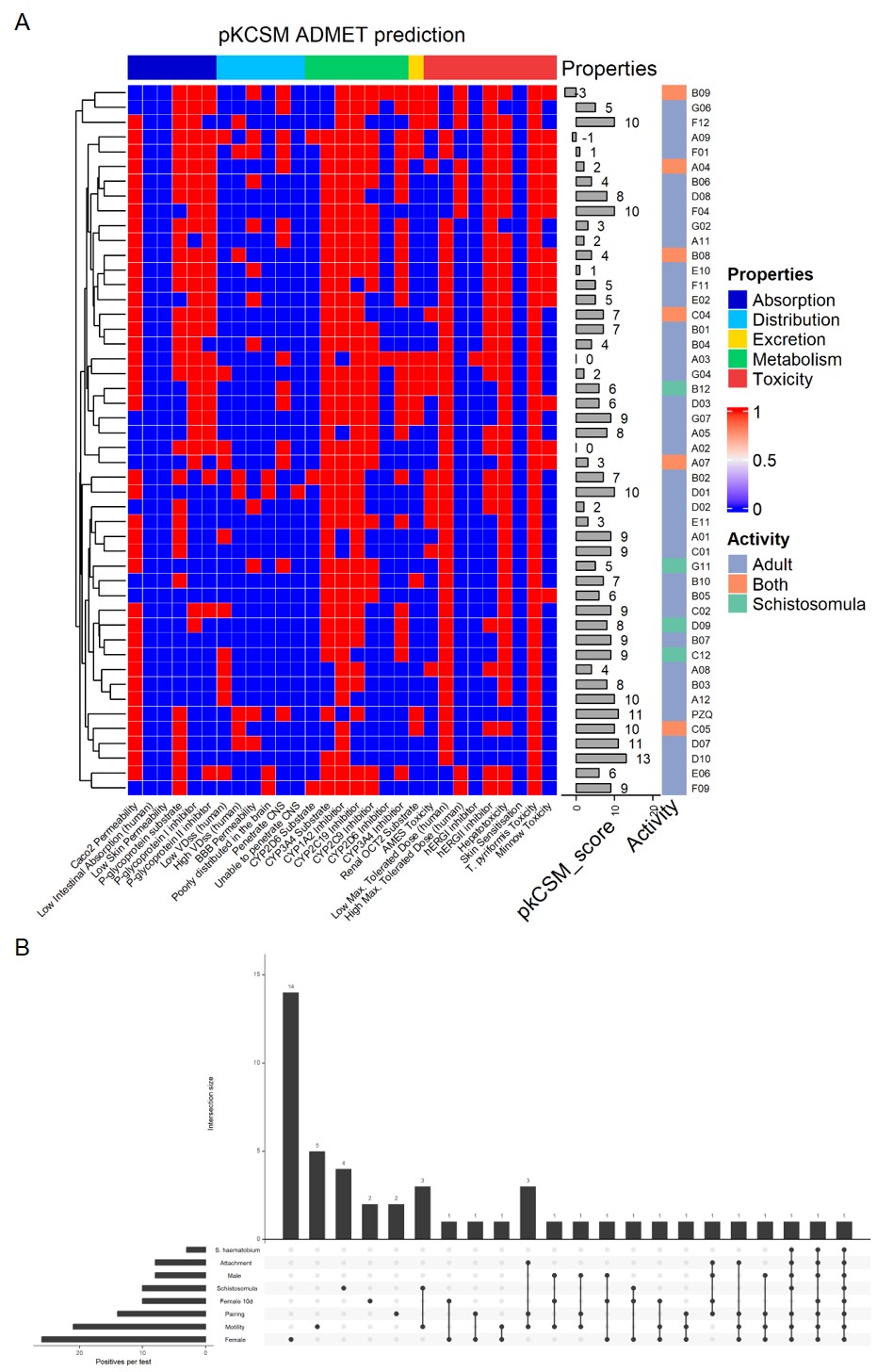


**Supplementary Figure S7.** Druglikeness of active compounds. (A) Hierarchical clustering of ADMET properties predicted using pkCSM web server. Red squares indicate the presence of features desired for good druggability, while blue squares represent the lack of the feature. Column sidebar colours indicate the following ADMET properties: Absorption (blue), Distribution (light blue), Metabolism (green), Excretion (yellow), and Toxicity (red). The row sidebar colours indicate whether the compounds presented activity against adult worms (lavender), schistosomula (light green) or both (orange). (B) Upset plot depicting the distribution of active compounds across different stages, sexes, or phenotypes evaluated. The plot highlights intersections, showcasing that some compounds exhibited activity in multiple evaluated conditions.

**
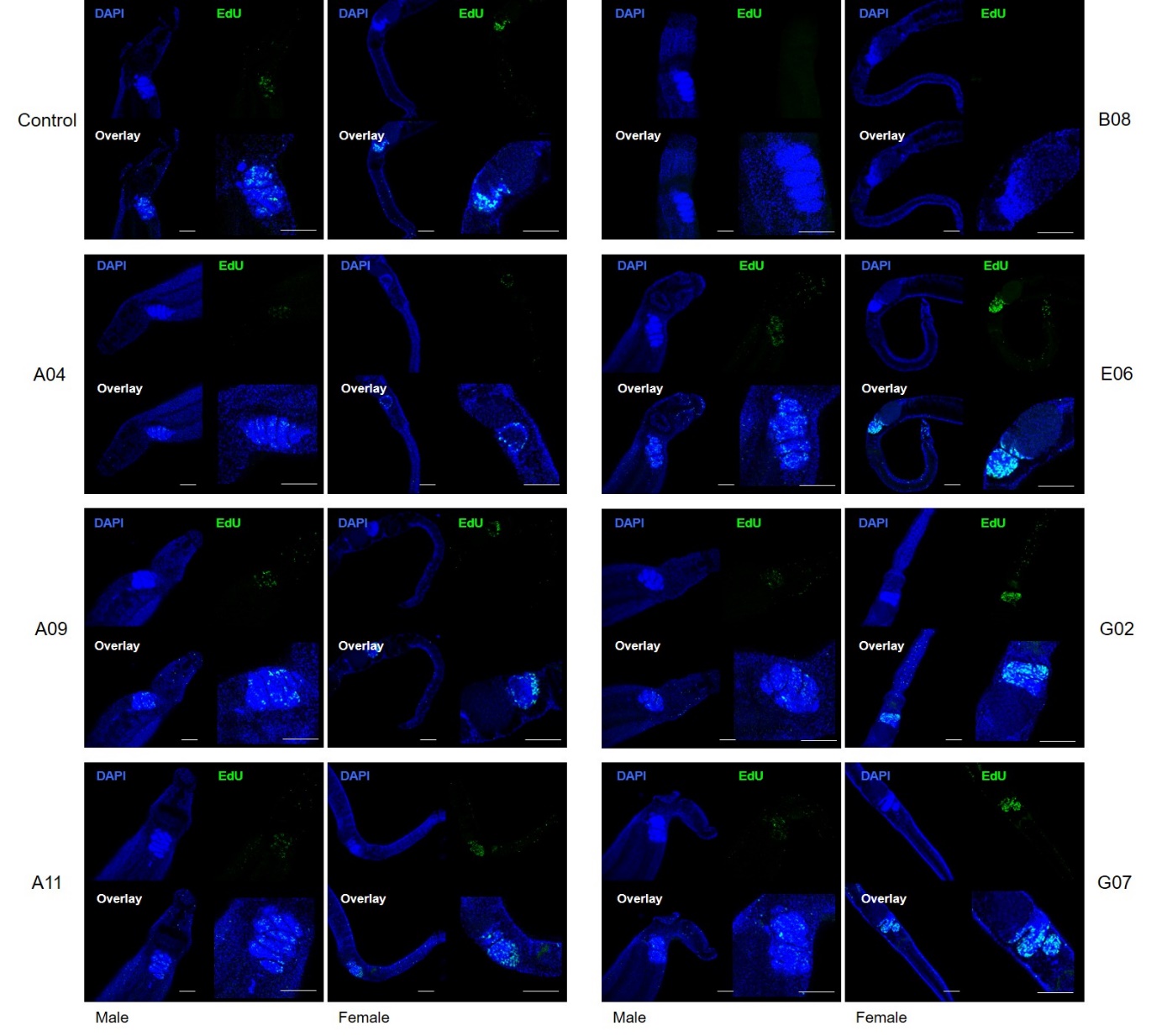
**

**Supplementary Figure S8.** Cell proliferation in adult worms. Couples of *S. mansoni* exposed to the compounds (A04, A09, A11, B08, E06, G02, and G07) with the highest antischistosomal activity were evaluated by EdU incorporation assay. Representative CLSM images from at least three couples per treatment are shown (green: proliferating cells; blue: cell nuclei). Scale bar represents 100 µm (overview) or 50 µm (magnification of gonads).


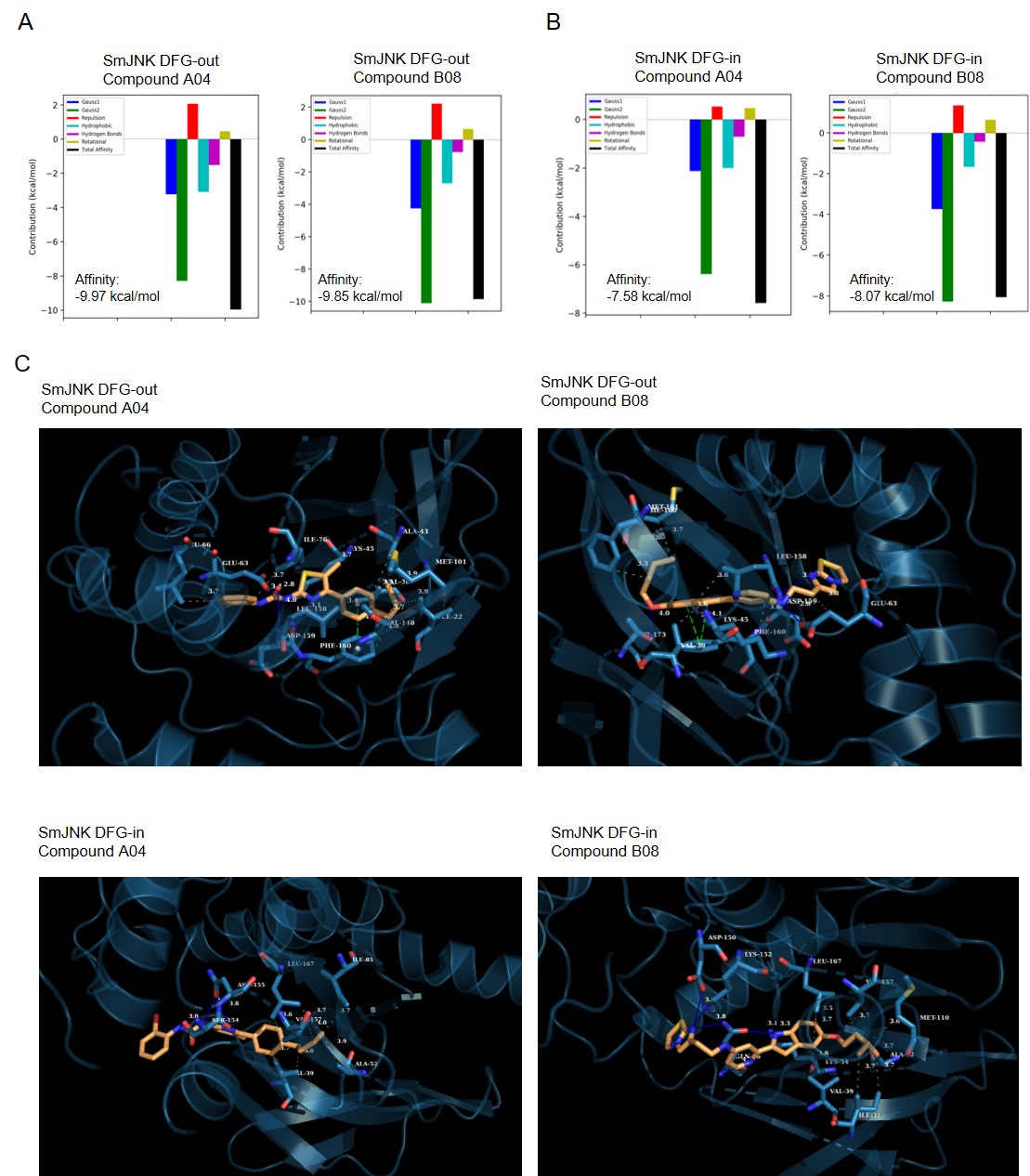


**Supplementary Figure S9.** Detailed analysis of the interaction of SmJNK in the active and inactive kinase conformation with compounds A04 and B08. A) Compounds A04 and B08 were predicted to block the SmJNK cavity formed in the inactive DFG-out kinase conformation (strongest affinity) through multiple multi-modal interactions with several flanking amino acid residues and a total binding affinity of −9.97 kcal/mol and −9.85 kcal/mol, respectively. B) Compounds A04 and B08 were predicted to bind to the SmJNK ATP pocket in the active kinase conformation (DFG in) through multiple multi-modal interactions with several flanking amino acid residues and a total binding affinity of −7.58 kcal/mol and −8.07 kcal/mol, respectively. C) Detailed interactions of (A) and (B) shown as rendered structure illustration of SmJNK (blue) and ligand (orange), with residues and distances designation.


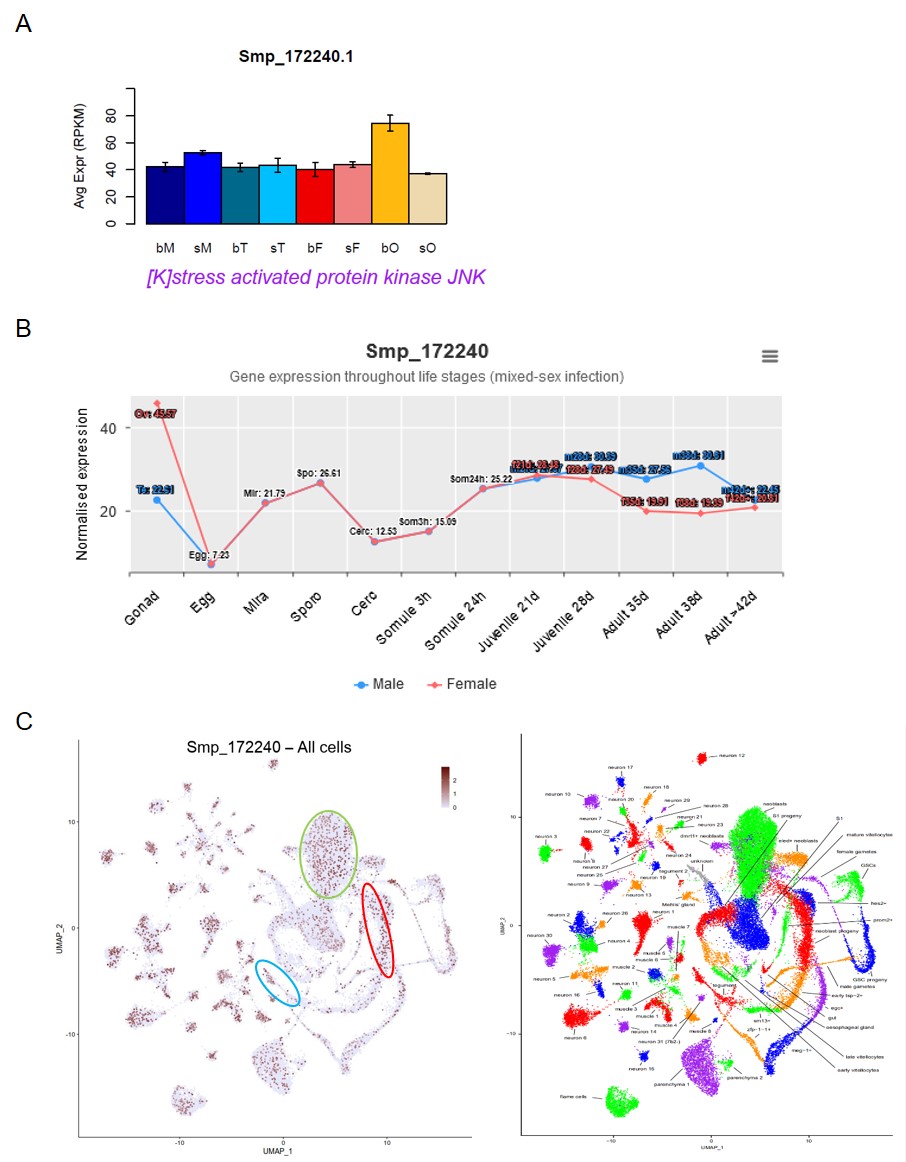


**Supplementary Figure S10. Graphical RNA-seq plots of SmJNK (Smp_172240) expression.** (A) Transcript profile of Smp_172240 according to a former study investigating the transcriptomes of adult *S. mansoni* and their gonads (Lu et al., 2016); bM, paired males; sM, unpaired males; bF, paired females; sF, unpaired females; bT and sT, testes of bM and sM, respectively; bO and sO, ovaries os bF and sF, respectively. (B) Results of a metaanalysis showing transcript profiles during the life cycle of *S. mansoni* as indicated (Lu et al., 2018). Of note, within the final host stages, SmJNK expression starts early (3 h) at the schistosomulum stage, increases within the first 24 h, and is then maintained during the further development of the adult stage at nearly equal levels in both genders; see also (A). (C) Single-cell atlas data showing a wide expression profile of SmJNK in many different tissues including the neoblasts (somatic stem cells; red circle left part) and a neoblast subpopulation (green circle), which leads to tegument progenitor cells and the tegument (blue circle) (Wendt et al., 2020).
